# Supplementary material for: Genomic Insights of Wheat Root-Associated Lysinibacillus fusiformis Reveal Its Related Functional Traits for Bioremediation of Soil Contaminated with Petroleum Products
Source: Microorganisms. 2024 Nov 20;12(11):2377. doi: 10.3390/microorganisms12112377 (PMC11596681; doi:10.3390/microorganisms12112377)
Supplement: Supplementary file 1 [file microorganisms-12-02377-s001.zip › microorganisms-3265211-supplementary.pdf]

Genomic Insights of Wheat Root-associated *Lysinibacillus fusiformis* reveal its Related Functional Traits for bioremediation of soil contaminated with petroleum products

Roderic Gilles Claret Diabankana[1*], Zhamalbekova Akerke Altaikyzy[2], Shakirova Aigerim Erbolkyzy[2], Valeriia Igorevna Vasiuk[1], Maria Nikolaevna Filimonova[3], Shamil Validov[1], Radik Ilyasovich Safin[4], Daniel Mawuena Afordoanyi[1,5*]

[1]Laboratory of Molecular Genetics and Microbiology Methods, Kazan Scientific Center Russian Academy of Sciences, 420111 Kazan, Russia.
[2] Kazakh Scientific Research Institute of Plant Protection and Quarantine named after Zh.Zhiembayev», Timiryazev St 45, Almaty 050040, Kazakhstan
[3]Academic and Research Centre, Institute of Fundamental Medicine and Biology, Kazan Federal University, 420008 Kazan, Russia
[4]Centre of Agroecological Research, Kazan State Agrarian University, 420015 Kazan, Russia
[5] Tatar Research Institute of Agriculture, Kazan Scientific Center of Russian Academy of Sciences, Kazan, Russia
*Correspondence: r.diabankana@knc.ru; d.afordoanyi@knc.ru

Table S1. Comparative analysis of a secondary metabolite profile of *Lysinibacillus fusiformis* strains Cu1-5, TZA38, HJ.T1, and MGMM7.

**Type**	**Most similar known cluster**	*L. fusiformis* Strains			
		**Cu1-5**	**HJ.T1**	**TZA38**	**MGMM7**
NRPS-like	Kijanimicin	(4%)	(4%)	(4%)	(4%)
T3PKS	Bacillibactin/bacillibactin E/bacillibactin F	(30%)	(30%)	(30%)	(30%)
Betalactone	Fengycin	(46%)	+ (46%)	(46%)	(46%)
Terpene		+	+	+	+
NI-siderophore	Petrobactin	(33%)	(33%)	(33%)	(33%)
Cyclic-lactone-autoinducer, LAP, thiopeptide, RRE-containing		+	+	+	-

Type	Most similar known cluster	*L. fusiformis* Strains			
		Cu1-5	HJ.T1	TZA38	MGMM7
Nrps	Molybdenum cofactor	-	-	-	(23%)
Ripp-like		-	+	-	-

N.B. «+» – gene present; «-» – gene absent; « %» – similarity percent to the most known cluster.

Table S2. KEGG mapper reconstruction comparison of *Lysinibacillus fusiformis* strains Cu1-5, TZA38, HJ.T1, and MGMM7.

KEGG Mapper Reconstruction	Gene's present (+) or absent (-) in genomes			
	TZA38	MGMM7	Cu15	HJT5
Benzoate degradation				
3-hydroxybutyryl-CoA dehydrogenase	+	+	+	+
acetyl-CoA C-acetyltransferase	+	+	+	+
acetyl-CoA acyltransferase	+	+	+	+
4-oxalocrotonate tautomerase	+	+	+	+
catechol 2,3-dioxygenase	+	+	+	+
3-hydroxyacyl-CoA dehydrogenase	+	+	+	+
enoyl-CoA hydratase	+	+	+	+
Aminobenzoate degradation				
acylphosphatase	+	+	+	+
amidase	-	+	+	-
Chloroalkane and chloroalkene degradation				
aldehyde dehydrogenase (NAD+)	+	+	+	+

2-haloacid dehalogenase	+	+	+	+
alcohol dehydrogenase	+	+	+	+
S-(hydroxymethyl)glutathione dehydrogenase	+	+	+	+
Chlorocyclohexane and chlorobenzene degradation				
2-haloacid dehalogenase	+	+	+	+
catechol 2,3-dioxygenase	+	+	+	+
Xylene degradation				
4-oxalocrotonate tautomerase	+	+	+	+
catechol 2,3-dioxygenase	+	+	+	+
Ethylbenzene degradation				
acetyl-CoA acyltransferase	+	+	+	+
Styrene degradation				
catechol 2,3-dioxygenase	+	+	+	+
amidase	-	+	+	-
Atrazine degradation				
urease subunit alpha, beta, and gamma	+	+	+	+
Caprolactam degradation				
cyclopentanol dehydrogenase	+	+	+	+
Dioxin degradation				
4-oxalocrotonate tautomerase	+	+	+	+
Naphthalene degradation				
Alcohol dehydrogenase	+	+	+	+

N-ethylmaleimide reductase				
N-ethylmaleimide reductase	+	+	+	+
S-(hydroxymethyl)glutathione dehydrogenase	-	+	+	+
Steroid degradation				
3-oxosteroid 1-dehydrogenase	+	+	+	+
**Degradation of aromatic compounds**				
4-hydroxyphenylacetate 3-monooxygenase	+	+	+	+
4-oxalocrotonate tautomerase	+	+	+	+
catechol 2,3-dioxygenase	+	+	+	+
alcohol dehydrogenase	+	+	+	+
cyclopentanol dehydrogenase	+	+	+	+
S-(hydroxymethyl)glutathione dehydrogenase	-	+	+	+
Ring-cleaving dioxygenase	+	+	+	+
Toluenesulfonate zinc-independent alcohol dehydrogenase	+	+	+	+
3-dehydroquinate dehydratase II	+	+	+	+
	+	+	+	+
hypoxanthine phosphoribosyltransferase	+	+	+	+
thymidine kinase	+	+	+	+
uridine kinase	+	+	+	+
dihydropyrimidinase	+	+	+	+
XTP/dITP diphosphohydrolase	+	+	+	+

N.B. «+» – gene present; «-» – gene absent.

Table S3. Genomic islands (GIs) of analyzed *L. fusiformis* strain Cu1-5, TZA38, HJ.T1, and MGMM7.

Lysinibacillus fusiformis strain HJ.T1.								
**Island start**	**Island end**	**Length**	**Gene ID**	**Locus**	**Gene start**	**Gene end**	**Strand**	**Product**
605213	635687	30474		DKHLHAKE_00574	605213	606277	1	ISL3 family transposase IS652
605213	635687	30474		DKHLHAKE_00575	606398	608266	1	hypothetical protein
605213	635687	30474		DKHLHAKE_00576	608610	613538	1	hypothetical protein
605213	635687	30474		DKHLHAKE_00577	613557	614153	1	hypothetical protein
605213	635687	30474		DKHLHAKE_00578	614424	614759	-1	hypothetical protein
605213	635687	30474		DKHLHAKE_00579	615306	615707	1	hypothetical protein
605213	635687	30474		DKHLHAKE_00580	615727	615861	1	hypothetical protein
605213	635687	30474		DKHLHAKE_00581	616030	616245	1	hypothetical protein
605213	635687	30474		DKHLHAKE_00582	616345	616683	1	hypothetical protein
605213	635687	30474	pld1_1	DKHLHAKE_00583	617272	618282	1	Pyridoxal 4-dehydrogenase
605213	635687	30474		DKHLHAKE_00584	618300	618710	1	hypothetical protein
605213	635687	30474	qacA	DKHLHAKE_00585	619084	620634	-1	Antiseptic resistance protein
605213	635687	30474	qacR_1	DKHLHAKE_00586	620846	621403	1	HTH-type transcriptional regulator QacR
605213	635687	30474		DKHLHAKE_00587	621901	622881	1	hypothetical protein
605213	635687	30474		DKHLHAKE_00588	623113	623637	1	IS3 family transposase ISBth167
605213	635687	30474		DKHLHAKE_00589	623625	624476	1	IS3 family transposase ISBce15
605213	635687	30474		DKHLHAKE_00590	624665	625168	1	IS1595 family transposase ISBko1
605213	635687	30474		DKHLHAKE_00591	626011	626700	-1	hypothetical protein
605213	635687	30474		DKHLHAKE_00592	626915	627751	1	Metallo-beta-lactamase type 2
605213	635687	30474	yxaF	DKHLHAKE_00593	628021	628620	1	putative HTH-type transcriptional regulator YxaF
605213	635687	30474	pbpX	DKHLHAKE_00594	628753	629733	1	Putative penicillin-binding protein PbpX
605213	635687	30474		DKHLHAKE_00595	631188	633836	1	hypothetical protein
605213	635687	30474		DKHLHAKE_00596	634790	634990	1	hypothetical protein
605213	635687	30474		DKHLHAKE_00597	635541	635687	1	hypothetical protein
1412356	1417417	5061		DKHLHAKE_01327	1412356	1413588	-1	hypothetical protein
1412356	1417417	5061		DKHLHAKE_01328	1413635	1414837	-1	hypothetical protein
1412356	1417417	5061		DKHLHAKE_01329	1414853	1416073	-1	hypothetical protein
1412356	1417417	5061		DKHLHAKE_01330	1416074	1417417	-1	hypothetical protein
2018938	2032791	13853		DKHLHAKE_01944	2018938	2019498	1	hypothetical protein
2018938	2032791	13853	cspB_2	DKHLHAKE_01945	2019802	2020002	1	Cold shock protein CspB
2018938	2032791	13853		DKHLHAKE_01946	2020196	2020423	1	hypothetical protein
2018938	2032791	13853		DKHLHAKE_01947	2020625	2020747	-1	hypothetical protein
2018938	2032791	13853		DKHLHAKE_01948	2020867	2021073	1	hypothetical protein
2018938	2032791	13853		DKHLHAKE_01949	2021921	2022115	1	hypothetical protein

2018938	2032791	13853		DKHLHAKE_01950	2022297	2022824	-1	hypothetical protein
2018938	2032791	13853	btuD_8	DKHLHAKE_01951	2023423	2024700	1	Vitamin B12 import ATP-binding protein BtuD
2018938	2032791	13853		DKHLHAKE_01952	2024700	2025707	1	hypothetical protein
2018938	2032791	13853		DKHLHAKE_01953	2025710	2025907	-1	hypothetical protein
2018938	2032791	13853	intQ	DKHLHAKE_01954	2025980	2027155	-1	Putative defective protein IntQ
2018938	2032791	13853		DKHLHAKE_01955	2027155	2027568	-1	hypothetical protein
2018938	2032791	13853		DKHLHAKE_01956	2027628	2028077	-1	hypothetical protein
2018938	2032791	13853		DKHLHAKE_01957	2028161	2029657	-1	hypothetical protein
2018938	2032791	13853		DKHLHAKE_01958	2029734	2029904	-1	hypothetical protein
2018938	2032791	13853		DKHLHAKE_01959	2030050	2030982	-1	hypothetical protein
2018938	2032791	13853	xre	DKHLHAKE_01960	2031286	2031642	-1	HTH-type transcriptional regulator Xre
2018938	2032791	13853		DKHLHAKE_01961	2031807	2032001	1	hypothetical protein
2018938	2032791	13853		DKHLHAKE_01962	2032070	2032402	-1	hypothetical protein
2018938	2032791	13853		DKHLHAKE_01963	2032483	2032791	1	hypothetical protein
2068490	2072848	4358		DKHLHAKE_01996	2068501	2068713	1	hypothetical protein
2068490	2072848	4358	yfiY_4	DKHLHAKE_01997	2069369	2070367	1	putative siderophore-binding lipoprotein YfiY
2068490	2072848	4358	rhaS_6	DKHLHAKE_01998	2070909	2072498	1	HTH-type transcriptional activator RhaS
2068490	2072848	4358		DKHLHAKE_01999	2072789	2073082	-1	hypothetical protein
2135331	2156046	20715		DKHLHAKE_02076	2135331	2136197	1	hypothetical protein
2135331	2156046	20715		DKHLHAKE_02077	2136537	2136965	-1	hypothetical protein
2135331	2156046	20715		DKHLHAKE_02078	2137098	2137505	-1	hypothetical protein
2135331	2156046	20715		DKHLHAKE_02079	2137912	2138121	1	hypothetical protein
2135331	2156046	20715	xerD_3	DKHLHAKE_02080	2138938	2139852	1	Tyrosine recombinase XerD
2135331	2156046	20715		DKHLHAKE_02081	2140562	2141194	1	hypothetical protein
2135331	2156046	20715		DKHLHAKE_02082	2141409	2142281	1	hypothetical protein
2135331	2156046	20715		DKHLHAKE_02083	2142908	2143129	-1	hypothetical protein
2135331	2156046	20715		DKHLHAKE_02084	2143284	2143541	1	hypothetical protein
2135331	2156046	20715		DKHLHAKE_02085	2144561	2144956	1	hypothetical protein
2135331	2156046	20715		DKHLHAKE_02086	2144953	2145123	1	hypothetical protein
2135331	2156046	20715		DKHLHAKE_02087	2145167	2145661	1	hypothetical protein
2135331	2156046	20715		DKHLHAKE_02088	2145905	2146819	1	hypothetical protein
2135331	2156046	20715		DKHLHAKE_02089	2146899	2147135	1	hypothetical protein
2135331	2156046	20715		DKHLHAKE_02090	2147176	2147430	1	hypothetical protein
2135331	2156046	20715		DKHLHAKE_02091	2147568	2147762	1	hypothetical protein
2135331	2156046	20715		DKHLHAKE_02092	2147759	2148217	1	hypothetical protein
2135331	2156046	20715		DKHLHAKE_02093	2148217	2149311	1	hypothetical protein
2135331	2156046	20715		DKHLHAKE_02094	2149594	2149896	1	hypothetical protein
2135331	2156046	20715		DKHLHAKE_02095	2150016	2150339	1	hypothetical protein
2135331	2156046	20715		DKHLHAKE_02096	2150330	2152510	1	hypothetical protein
2135331	2156046	20715		DKHLHAKE_02097	2152698	2153210	1	hypothetical protein
2135331	2156046	20715		DKHLHAKE_02098	2153195	2153599	1	hypothetical protein

2135331	2156046	20715		DKHLHAKE_02099	2154456	2154761	1	hypothetical protein
2135331	2156046	20715		DKHLHAKE_02100	2155288	2156046	1	hypothetical protein
2137098	2141194	4096		DKHLHAKE_02078	2137098	2137505	-1	hypothetical protein
2137098	2141194	4096		DKHLHAKE_02079	2137912	2138121	1	hypothetical protein
2137098	2141194	4096	xerD_3	DKHLHAKE_02080	2138938	2139852	1	Tyrosine recombinase XerD
2137098	2141194	4096		DKHLHAKE_02081	2140562	2141194	1	hypothetical protein
2336106	2340419	4313		DKHLHAKE_02273	2336106	2336336	1	hypothetical protein
2336106	2340419	4313	gloB_4	DKHLHAKE_02274	2336917	2337939	-1	Hydroxyacylglutathione hydrolase
2336106	2340419	4313		DKHLHAKE_02275	2338106	2339116	1	hypothetical protein
2336106	2340419	4313	slmA_3	DKHLHAKE_02276	2339211	2339786	1	Nucleoid occlusion factor SlmA
2336106	2340419	4313		DKHLHAKE_02277	2340225	2340419	-1	hypothetical protein
2345141	2386803	41662	menH_2	DKHLHAKE_02282	2345141	2346982	1	2-succinyl-6-hydroxy-2, 4-cyclohexadiene-1-carboxylate synthase
2345141	2386803	41662	yflT_2	DKHLHAKE_02283	2347419	2347739	-1	General stress protein 17M
2345141	2386803	41662		DKHLHAKE_02284	2348246	2348950	1	hypothetical protein
2345141	2386803	41662		DKHLHAKE_02285	2349170	2349580	1	hypothetical protein
2345141	2386803	41662		DKHLHAKE_02286	2349959	2350504	1	hypothetical protein
2345141	2386803	41662		DKHLHAKE_02287	2350655	2351218	1	hypothetical protein
2345141	2386803	41662	pld1_2	DKHLHAKE_02288	2351718	2352728	1	Pyridoxal 4-dehydrogenase
2345141	2386803	41662		DKHLHAKE_02289	2352746	2353156	1	hypothetical protein
2345141	2386803	41662	betI_9	DKHLHAKE_02290	2353482	2354066	1	HTH-type transcriptional regulator BetI
2345141	2386803	41662		DKHLHAKE_02291	2354063	2354815	1	hypothetical protein
2345141	2386803	41662		DKHLHAKE_02292	2354995	2355585	1	hypothetical protein
2345141	2386803	41662	rppH_2	DKHLHAKE_02293	2355757	2356167	1	RNA pyrophosphohydrolase
2345141	2386803	41662		DKHLHAKE_02294	2356258	2356782	1	IS3 family transposase ISBth167
2345141	2386803	41662		DKHLHAKE_02295	2356770	2357621	1	IS3 family transposase ISBce15
2345141	2386803	41662	xerC_3	DKHLHAKE_02296	2357926	2358642	1	Tyrosine recombinase XerC
2345141	2386803	41662		DKHLHAKE_02297	2358826	2359713	1	hypothetical protein
2345141	2386803	41662		DKHLHAKE_02298	2360267	2360518	1	hypothetical protein
2345141	2386803	41662		DKHLHAKE_02299	2360865	2361269	1	hypothetical protein
2345141	2386803	41662		DKHLHAKE_02300	2361519	2362712	-1	hypothetical protein
2345141	2386803	41662		DKHLHAKE_02301	2362942	2363409	-1	hypothetical protein
2345141	2386803	41662	ant1	DKHLHAKE_02302	2363620	2363922	1	Streptomycin 3''-adenylyltransferase
2345141	2386803	41662		DKHLHAKE_02303	2364049	2364333	1	hypothetical protein
2345141	2386803	41662		DKHLHAKE_02304	2364653	2365420	1	hypothetical protein
2345141	2386803	41662	accD_1	DKHLHAKE_02305	2366708	2367556	1	Acetyl-coenzyme A carboxylase carboxyl transferase subunit beta
2345141	2386803	41662	accA_1	DKHLHAKE_02306	2367549	2368505	1	Acetyl-coenzyme A carboxylase carboxyl transferase subunit alpha
2345141	2386803	41662	recF_3	DKHLHAKE_02307	2370310	2372274	1	DNA replication and repair protein RecF
2345141	2386803	41662	rep	DKHLHAKE_02308	2372282	2374237	1	ATP-dependent DNA helicase Rep
2345141	2386803	41662		DKHLHAKE_02309	2375128	2377128	1	hypothetical protein
2345141	2386803	41662		DKHLHAKE_02310	2378496	2383046	1	hypothetical protein
2345141	2386803	41662		DKHLHAKE_02311	2383992	2384810	-1	hypothetical protein

2345141	2386803	41662	adaB_1	DKHLHAKE_02312	2384847	2385380	-1	Methylated-DNA-protein-cysteine methyltransferase, inducible
2345141	2386803	41662	adaA_2	DKHLHAKE_02313	2385364	2386002	-1	Bifunctional transcriptional activator/DNA repair enzyme AdaA
2345141	2386803	41662		DKHLHAKE_02314	2386090	2386803	-1	hypothetical protein
2466554	2470665	4111		DKHLHAKE_02394	2466814	2467938	-1	hypothetical protein
2466554	2470665	4111		DKHLHAKE_02395	2468298	2468822	1	IS3 family transposase ISBth167
2466554	2470665	4111		DKHLHAKE_02396	2468810	2469661	1	IS3 family transposase ISBce15
2466554	2470665	4111		DKHLHAKE_02397	2469738	2470406	-1	hypothetical protein
2675498	2691983	16485		DKHLHAKE_02625	2675498	2677156	-1	hypothetical protein
2675498	2691983	16485		DKHLHAKE_02626	2677217	2678278	-1	hypothetical protein
2675498	2691983	16485		DKHLHAKE_02627	2678529	2679077	-1	hypothetical protein
2675498	2691983	16485		DKHLHAKE_02628	2679550	2679993	1	hypothetical protein
2675498	2691983	16485	ktrA_3	DKHLHAKE_02629	2680110	2680400	-1	Ktr system potassium uptake protein A
2675498	2691983	16485		DKHLHAKE_02630	2680491	2680928	-1	hypothetical protein
2675498	2691983	16485		DKHLHAKE_02631	2680950	2681627	-1	hypothetical protein
2675498	2691983	16485		DKHLHAKE_02632	2682082	2683116	-1	hypothetical protein
2675498	2691983	16485		DKHLHAKE_02633	2683800	2683946	-1	hypothetical protein
2675498	2691983	16485		DKHLHAKE_02634	2683946	2684152	-1	hypothetical protein
2675498	2691983	16485		DKHLHAKE_02635	2684210	2684338	-1	hypothetical protein
2675498	2691983	16485		DKHLHAKE_02636	2684638	2685063	-1	hypothetical protein
2675498	2691983	16485		DKHLHAKE_02637	2685719	2685979	-1	hypothetical protein
2675498	2691983	16485		DKHLHAKE_02638	2686148	2686513	-1	hypothetical protein
2675498	2691983	16485		DKHLHAKE_02639	2686809	2687174	-1	hypothetical protein
2675498	2691983	16485		DKHLHAKE_02640	2687257	2687472	-1	hypothetical protein
2675498	2691983	16485		DKHLHAKE_02641	2687628	2687789	-1	hypothetical protein
2675498	2691983	16485	ydaD	DKHLHAKE_02642	2687875	2688876	1	General stress protein 39
2675498	2691983	16485		DKHLHAKE_02643	2689144	2689701	-1	IS1595 family transposase ISSpgl1
2675498	2691983	16485		DKHLHAKE_02644	2689807	2690106	-1	hypothetical protein
2675498	2691983	16485		DKHLHAKE_02645	2690379	2690603	1	hypothetical protein
2675498	2691983	16485		DKHLHAKE_02646	2691136	2691414	1	hypothetical protein
2675498	2691983	16485		DKHLHAKE_02647	2691507	2691983	1	hypothetical protein
2966874	3007158	40284		DKHLHAKE_02916	2966874	2967257	-1	hypothetical protein
2966874	3007158	40284	tri1	DKHLHAKE_02917	2967284	2968231	-1	ADP-ribosylarginine hydrolase Tri1
2966874	3007158	40284		DKHLHAKE_02918	2969145	2969447	-1	hypothetical protein
2966874	3007158	40284		DKHLHAKE_02919	2969643	2970446	-1	hypothetical protein
2966874	3007158	40284		DKHLHAKE_02920	2970563	2971069	-1	hypothetical protein
2966874	3007158	40284		DKHLHAKE_02921	2971299	2971445	-1	hypothetical protein
2966874	3007158	40284		DKHLHAKE_02922	2971545	2971808	-1	hypothetical protein
2966874	3007158	40284		DKHLHAKE_02923	2971932	2972312	-1	hypothetical protein
2966874	3007158	40284		DKHLHAKE_02924	2973109	2973489	-1	hypothetical protein
2966874	3007158	40284		DKHLHAKE_02925	2973768	2974193	-1	hypothetical protein
2966874	3007158	40284		DKHLHAKE_02926	2974345	2974728	-1	hypothetical protein

2966874	3007158	40284		DKHLHAKE_02927	2975126	2975410	-1	hypothetical protein
2966874	3007158	40284	nfi_1	DKHLHAKE_02928	2976085	2976324	-1	Endonuclease V
2966874	3007158	40284	nfi_2	DKHLHAKE_02929	2976317	2976760	-1	Endonuclease V
2966874	3007158	40284		DKHLHAKE_02930	2976781	2977209	-1	hypothetical protein
2966874	3007158	40284		DKHLHAKE_02931	2977736	2978497	-1	hypothetical protein
2966874	3007158	40284		DKHLHAKE_02932	2979223	2979609	-1	hypothetical protein
2966874	3007158	40284		DKHLHAKE_02933	2979606	2979908	-1	hypothetical protein
2966874	3007158	40284		DKHLHAKE_02934	2980547	2981341	-1	hypothetical protein
2966874	3007158	40284		DKHLHAKE_02935	2982456	2983184	-1	hypothetical protein
2966874	3007158	40284		DKHLHAKE_02936	2983188	2983820	-1	hypothetical protein
2966874	3007158	40284		DKHLHAKE_02937	2984091	2984183	-1	hypothetical protein
2966874	3007158	40284		DKHLHAKE_02938	2984454	2984804	-1	hypothetical protein
2966874	3007158	40284	essG	DKHLHAKE_02939	2985376	2985855	-1	Type VII secretion system protein EsaG
2966874	3007158	40284	yeeF	DKHLHAKE_02940	2985860	2986168	-1	Putative ribonuclease YeeF
2966874	3007158	40284		DKHLHAKE_02941	2986770	2987072	-1	hypothetical protein
2966874	3007158	40284		DKHLHAKE_02942	2987097	2987393	-1	hypothetical protein
2966874	3007158	40284		DKHLHAKE_02943	2987652	2988233	-1	hypothetical protein
2966874	3007158	40284		DKHLHAKE_02944	2988371	2989066	-1	hypothetical protein
2966874	3007158	40284		DKHLHAKE_02945	2989081	2990463	-1	hypothetical protein
2966874	3007158	40284		DKHLHAKE_02946	2990470	2990820	-1	hypothetical protein
2966874	3007158	40284		DKHLHAKE_02947	2991107	2991631	-1	hypothetical protein
2966874	3007158	40284		DKHLHAKE_02948	2991831	2992214	-1	hypothetical protein
2966874	3007158	40284		DKHLHAKE_02949	2992518	2992901	-1	hypothetical protein
2966874	3007158	40284		DKHLHAKE_02950	2992916	2993725	-1	hypothetical protein
2966874	3007158	40284		DKHLHAKE_02951	2994118	2994570	-1	hypothetical protein
2966874	3007158	40284		DKHLHAKE_02952	2994595	2995572	-1	hypothetical protein
2966874	3007158	40284		DKHLHAKE_02953	2995659	2995982	-1	hypothetical protein
2966874	3007158	40284		DKHLHAKE_02954	2995994	2996374	-1	hypothetical protein
2966874	3007158	40284		DKHLHAKE_02955	2996364	2996669	-1	hypothetical protein
2966874	3007158	40284		DKHLHAKE_02956	2996644	2997012	-1	hypothetical protein
2966874	3007158	40284		DKHLHAKE_02957	2998304	2998732	-1	hypothetical protein
2966874	3007158	40284	cdiA	DKHLHAKE_02958	2998698	2999045	-1	tRNA nuclease CdiA
2966874	3007158	40284		DKHLHAKE_02959	2999204	3000442	-1	IS110 family transposase ISCth6
2966874	3007158	40284		DKHLHAKE_02960	3000591	3001484	-1	hypothetical protein
2966874	3007158	40284		DKHLHAKE_02961	3001580	3001795	1	hypothetical protein
2966874	3007158	40284		DKHLHAKE_02962	3001792	3002613	-1	hypothetical protein
2966874	3007158	40284		DKHLHAKE_02963	3003340	3003591	-1	hypothetical protein
2966874	3007158	40284		DKHLHAKE_02964	3003661	3004170	-1	hypothetical protein
2966874	3007158	40284		DKHLHAKE_02965	3004175	3005629	-1	hypothetical protein
2966874	3007158	40284		DKHLHAKE_02966	3005629	3007158	-1	hypothetical protein
3395848	3418441	22593		DKHLHAKE_03356	3395848	3396291	-1	hypothetical protein

3395848	3418441	22593		DKHLHAKE_03357	3396288	3396704	-1	hypothetical protein
3395848	3418441	22593		DKHLHAKE_03358	3396704	3397024	-1	hypothetical protein
3395848	3418441	22593		DKHLHAKE_03359	3397011	3397337	-1	hypothetical protein
3395848	3418441	22593		DKHLHAKE_03360	3397346	3397504	-1	hypothetical protein
3395848	3418441	22593		DKHLHAKE_03361	3397518	3398381	-1	hypothetical protein
3395848	3418441	22593		DKHLHAKE_03362	3398412	3399074	-1	hypothetical protein
3395848	3418441	22593		DKHLHAKE_03363	3399281	3399502	-1	hypothetical protein
3395848	3418441	22593		DKHLHAKE_03364	3399647	3400663	-1	hypothetical protein
3395848	3418441	22593		DKHLHAKE_03365	3400660	3402078	-1	hypothetical protein
3395848	3418441	22593		DKHLHAKE_03366	3402091	3403353	-1	hypothetical protein
3395848	3418441	22593		DKHLHAKE_03367	3403353	3404141	-1	hypothetical protein
3395848	3418441	22593		DKHLHAKE_03368	3404214	3404420	-1	hypothetical protein
3395848	3418441	22593		DKHLHAKE_03369	3404660	3405166	-1	hypothetical protein
3395848	3418441	22593		DKHLHAKE_03370	3405381	3405569	1	hypothetical protein
3395848	3418441	22593		DKHLHAKE_03371	3405601	3406017	-1	hypothetical protein
3395848	3418441	22593		DKHLHAKE_03372	3406531	3407529	1	hypothetical protein
3395848	3418441	22593		DKHLHAKE_03373	3407538	3407972	-1	hypothetical protein
3395848	3418441	22593		DKHLHAKE_03374	3408302	3409027	1	hypothetical protein
3395848	3418441	22593		DKHLHAKE_03375	3409262	3409450	-1	hypothetical protein
3395848	3418441	22593		DKHLHAKE_03376	3409443	3409631	-1	hypothetical protein
3395848	3418441	22593		DKHLHAKE_03377	3409666	3410418	-1	hypothetical protein
3395848	3418441	22593		DKHLHAKE_03378	3410471	3411337	-1	hypothetical protein
3395848	3418441	22593		DKHLHAKE_03379	3411353	3411472	-1	hypothetical protein
3395848	3418441	22593		DKHLHAKE_03380	3411632	3411829	1	hypothetical protein
3395848	3418441	22593		DKHLHAKE_03381	3411850	3412026	-1	hypothetical protein
3395848	3418441	22593		DKHLHAKE_03382	3412023	3412562	-1	hypothetical protein
3395848	3418441	22593		DKHLHAKE_03383	3412581	3413384	-1	hypothetical protein
3395848	3418441	22593		DKHLHAKE_03384	3413335	3414234	-1	hypothetical protein
3395848	3418441	22593		DKHLHAKE_03385	3414304	3414534	-1	hypothetical protein
3395848	3418441	22593		DKHLHAKE_03386	3414531	3414740	-1	hypothetical protein
3395848	3418441	22593		DKHLHAKE_03387	3415052	3415294	-1	hypothetical protein
3395848	3418441	22593		DKHLHAKE_03388	3415291	3415809	-1	hypothetical protein
3395848	3418441	22593		DKHLHAKE_03389	3415991	3416341	-1	hypothetical protein
3395848	3418441	22593		DKHLHAKE_03390	3416383	3416610	-1	hypothetical protein
3395848	3418441	22593		DKHLHAKE_03391	3416625	3416783	-1	hypothetical protein
3395848	3418441	22593		DKHLHAKE_03392	3416851	3417648	-1	hypothetical protein
3395848	3418441	22593		DKHLHAKE_03393	3417716	3417991	1	hypothetical protein
3395848	3418441	22593		DKHLHAKE_03394	3417945	3418160	-1	hypothetical protein
3395848	3418441	22593		DKHLHAKE_03395	3418214	3418441	-1	hypothetical protein
3427283	3432956	5673	murI	DKHLHAKE_03408	3427283	3428080	-1	Glutamate racemase
3427283	3432956	5673	slyA_4	DKHLHAKE_03409	3428091	3428543	-1	Transcriptional regulator SlyA

3427283	3432956	5673	gerE	DKHLHAKE_03410	3428618	3428839	-1	Spore germination protein GerE
3427283	3432956	5673		DKHLHAKE_03411	3429028	3429573	-1	hypothetical protein
3427283	3432956	5673	frdB	DKHLHAKE_03412	3429743	3430522	-1	Fumarate reductase iron-sulfur subunit
3427283	3432956	5673	frdA	DKHLHAKE_03413	3430528	3432282	-1	Fumarate reductase flavoprotein subunit
3427283	3432956	5673	sdhC	DKHLHAKE_03414	3432348	3432956	-1	Succinate dehydrogenase cytochrome b558 subunit
***Lysinibacillus fusiformis* strain TZA38.**								
**Island start**	**Island end**	**Length**	**Gene ID**	**Locus**	**Gene start**	**Gene end**	**Strand**	**Product**
467490	485283	17793	radA_1	EDBLDKDH_00478	466876	467514	-1	DNA repair protein RadA
467490	485283	17793	tmk_1	EDBLDKDH_00479	467745	468374	1	Thymidylate kinase
467490	485283	17793		EDBLDKDH_00480	468392	468637	1	hypothetical protein
467490	485283	17793		EDBLDKDH_00481	468651	469001	1	hypothetical protein
467490	485283	17793		EDBLDKDH_00482	469004	469954	1	hypothetical protein
467490	485283	17793		EDBLDKDH_00483	469965	470624	1	hypothetical protein
467490	485283	17793		EDBLDKDH_00484	470715	471383	1	hypothetical protein
467490	485283	17793		EDBLDKDH_00485	471395	472087	1	hypothetical protein
467490	485283	17793	thyA_1	EDBLDKDH_00486	472108	473067	1	Thymidylate synthase
467490	485283	17793		EDBLDKDH_00487	473193	474428	-1	hypothetical protein
467490	485283	17793		EDBLDKDH_00488	474586	475374	1	hypothetical protein
467490	485283	17793		EDBLDKDH_00489	475542	476063	-1	hypothetical protein
467490	485283	17793		EDBLDKDH_00490	476087	476494	-1	hypothetical protein
467490	485283	17793		EDBLDKDH_00491	476625	476930	1	hypothetical protein
467490	485283	17793	radA_2	EDBLDKDH_00492	477091	478476	-1	DNA repair protein RadA
467490	485283	17793		EDBLDKDH_00493	478495	479166	-1	hypothetical protein
467490	485283	17793		EDBLDKDH_00494	479159	480910	-1	hypothetical protein
467490	485283	17793		EDBLDKDH_00495	480915	483854	-1	hypothetical protein
467490	485283	17793		EDBLDKDH_00496	483861	485090	-1	hypothetical protein
467490	485283	17793	radA_3	EDBLDKDH_00497	485252	486103	-1	DNA repair protein RadA
1963566	1971455	7889		EDBLDKDH_01900	1963566	1964990	-1	hypothetical protein
1963566	1971455	7889	lexA_2	EDBLDKDH_01901	1965059	1965658	-1	LexA repressor
1963566	1971455	7889		EDBLDKDH_01902	1965821	1966060	1	hypothetical protein
1963566	1971455	7889		EDBLDKDH_01903	1966128	1966277	1	hypothetical protein
1963566	1971455	7889		EDBLDKDH_01904	1966330	1966431	1	hypothetical protein
1963566	1971455	7889		EDBLDKDH_01905	1966464	1966964	1	hypothetical protein
1963566	1971455	7889		EDBLDKDH_01906	1966936	1967238	-1	hypothetical protein
1963566	1971455	7889		EDBLDKDH_01907	1967300	1967782	1	hypothetical protein
1963566	1971455	7889		EDBLDKDH_01908	1967742	1967888	1	hypothetical protein
1963566	1971455	7889		EDBLDKDH_01909	1967903	1968613	1	hypothetical protein
1963566	1971455	7889	abrB_2	EDBLDKDH_01910	1968591	1968860	1	Transition state regulatory protein AbrB
1963566	1971455	7889		EDBLDKDH_01911	1968869	1969042	1	hypothetical protein
1963566	1971455	7889		EDBLDKDH_01912	1969045	1969518	1	hypothetical protein

1963566	1971455	7889		EDBLDKDH_01913	1969529	1969891	1	hypothetical protein
1963566	1971455	7889		EDBLDKDH_01914	1969905	1970237	1	hypothetical protein
1963566	1971455	7889		EDBLDKDH_01915	1970259	1970972	1	hypothetical protein
1963566	1971455	7889		EDBLDKDH_01916	1970976	1971455	1	hypothetical protein
2667904	2675522	7618	crp	EDBLDKDH_02633	2667835	2668467	-1	cAMP-activated global transcriptional regulator CRP
2667904	2675522	7618	rihA_1	EDBLDKDH_02634	2668561	2669514	1	Pyrimidine-specific ribonucleoside hydrolase RihA
2667904	2675522	7618	rbsK_1	EDBLDKDH_02635	2669532	2670416	1	Ribokinase
2667904	2675522	7618	puuR_2	EDBLDKDH_02636	2670704	2671252	1	HTH-type transcriptional regulator PuuR
2667904	2675522	7618	mvaA	EDBLDKDH_02637	2671392	2672648	1	3-hydroxy-3-methylglutaryl-coenzyme A reductase
2667904	2675522	7618		EDBLDKDH_02638	2672650	2673090	1	hypothetical protein
2667904	2675522	7618		EDBLDKDH_02639	2673087	2674442	1	hypothetical protein
2667904	2675522	7618	yngG_2	EDBLDKDH_02640	2674462	2675358	1	Hydroxymethylglutaryl-CoA lyase YngG
3150655	3157941	7286		EDBLDKDH_03110	3150655	3151053	1	hypothetical protein
3150655	3157941	7286		EDBLDKDH_03111	3151868	3152338	-1	hypothetical protein
3150655	3157941	7286		EDBLDKDH_03112	3152483	3153115	1	hypothetical protein
3150655	3157941	7286		EDBLDKDH_03113	3153520	3153783	-1	hypothetical protein
3150655	3157941	7286		EDBLDKDH_03114	3154181	3154441	1	hypothetical protein
3150655	3157941	7286		EDBLDKDH_03115	3154621	3155220	1	hypothetical protein
3150655	3157941	7286		EDBLDKDH_03116	3155771	3155893	1	hypothetical protein
3150655	3157941	7286		EDBLDKDH_03117	3156323	3156640	-1	hypothetical protein
3150655	3157941	7286		EDBLDKDH_03118	3156869	3157024	1	hypothetical protein
3150655	3157941	7286	xerH	EDBLDKDH_03119	3157465	3157941	1	Tyrosine recombinase XerH
3251074	3257016	5942		EDBLDKDH_03228	3251074	3251499	-1	hypothetical protein
3251074	3257016	5942		EDBLDKDH_03229	3251792	3252157	-1	hypothetical protein
3251074	3257016	5942		EDBLDKDH_03230	3252240	3252455	-1	hypothetical protein
3251074	3257016	5942		EDBLDKDH_03231	3252610	3252771	-1	hypothetical protein
3251074	3257016	5942	ydaD_2	EDBLDKDH_03232	3252857	3253858	1	General stress protein 39
3251074	3257016	5942		EDBLDKDH_03233	3254165	3254722	-1	IS1595 family transposase ISSpgl1
3251074	3257016	5942		EDBLDKDH_03234	3254843	3255124	-1	hypothetical protein
3251074	3257016	5942		EDBLDKDH_03235	3255405	3255629	1	hypothetical protein
3251074	3257016	5942		EDBLDKDH_03236	3256169	3256447	1	hypothetical protein
3251074	3257016	5942		EDBLDKDH_03237	3256540	3257016	1	hypothetical protein
3915314	3919395	4081		EDBLDKDH_03887	3915314	3915787	-1	hypothetical protein
3915314	3919395	4081		EDBLDKDH_03888	3915920	3916456	-1	hypothetical protein
3915314	3919395	4081		EDBLDKDH_03889	3916897	3918315	-1	hypothetical protein
3915314	3919395	4081		EDBLDKDH_03890	3918316	3918855	-1	hypothetical protein
3915314	3919395	4081		EDBLDKDH_03891	3918874	3919395	-1	hypothetical protein
3931923	3970965	39042		EDBLDKDH_03908	3931923	3932189	-1	hypothetical protein
3931923	3970965	39042		EDBLDKDH_03909	3932208	3932600	-1	hypothetical protein
3931923	3970965	39042		EDBLDKDH_03910	3932738	3933028	-1	hypothetical protein
3931923	3970965	39042		EDBLDKDH_03911	3933109	3933507	-1	hypothetical protein

3931923	3970965	39042		EDBLDKDH_03912	3933594	3933803	-1	hypothetical protein
3931923	3970965	39042		EDBLDKDH_03913	3933866	3934156	-1	hypothetical protein
3931923	3970965	39042		EDBLDKDH_03914	3934240	3934434	-1	hypothetical protein
3931923	3970965	39042		EDBLDKDH_03915	3934450	3936264	-1	hypothetical protein
3931923	3970965	39042		EDBLDKDH_03916	3936328	3937062	-1	hypothetical protein
3931923	3970965	39042		EDBLDKDH_03917	3937511	3937669	-1	hypothetical protein
3931923	3970965	39042		EDBLDKDH_03918	3937669	3937977	-1	hypothetical protein
3931923	3970965	39042		EDBLDKDH_03919	3937995	3939863	-1	hypothetical protein
3931923	3970965	39042		EDBLDKDH_03920	3939875	3940795	-1	hypothetical protein
3931923	3970965	39042		EDBLDKDH_03921	3940808	3945364	-1	hypothetical protein
3931923	3970965	39042		EDBLDKDH_03922	3945656	3946159	-1	hypothetical protein
3931923	3970965	39042		EDBLDKDH_03923	3946224	3946997	-1	hypothetical protein
3931923	3970965	39042		EDBLDKDH_03924	3947008	3947430	-1	hypothetical protein
3931923	3970965	39042		EDBLDKDH_03925	3947427	3947843	-1	hypothetical protein
3931923	3970965	39042		EDBLDKDH_03926	3947843	3948166	-1	hypothetical protein
3931923	3970965	39042		EDBLDKDH_03927	3948153	3948479	-1	hypothetical protein
3931923	3970965	39042		EDBLDKDH_03928	3948488	3948643	-1	hypothetical protein
3931923	3970965	39042		EDBLDKDH_03929	3948663	3949526	-1	hypothetical protein
3931923	3970965	39042		EDBLDKDH_03930	3949556	3950218	-1	hypothetical protein
3931923	3970965	39042		EDBLDKDH_03931	3950500	3950982	-1	hypothetical protein
3931923	3970965	39042		EDBLDKDH_03932	3951130	3952149	-1	hypothetical protein
3931923	3970965	39042		EDBLDKDH_03933	3952146	3953564	-1	hypothetical protein
3931923	3970965	39042		EDBLDKDH_03934	3953577	3954785	-1	hypothetical protein
3931923	3970965	39042		EDBLDKDH_03935	3954785	3955606	-1	hypothetical protein
3931923	3970965	39042		EDBLDKDH_03936	3955680	3955946	-1	hypothetical protein
3931923	3970965	39042		EDBLDKDH_03937	3955990	3956379	-1	hypothetical protein
3931923	3970965	39042		EDBLDKDH_03938	3956594	3956827	-1	hypothetical protein
3931923	3970965	39042		EDBLDKDH_03939	3957523	3958341	-1	hypothetical protein
3931923	3970965	39042		EDBLDKDH_03940	3958338	3958679	-1	hypothetical protein
3931923	3970965	39042		EDBLDKDH_03941	3958907	3959341	-1	hypothetical protein
3931923	3970965	39042		EDBLDKDH_03942	3959459	3959593	-1	hypothetical protein
3931923	3970965	39042		EDBLDKDH_03943	3959590	3959778	-1	hypothetical protein
3931923	3970965	39042		EDBLDKDH_03944	3959771	3959959	-1	hypothetical protein
3931923	3970965	39042		EDBLDKDH_03945	3959959	3960288	-1	hypothetical protein
3931923	3970965	39042		EDBLDKDH_03946	3960281	3960769	-1	hypothetical protein
3931923	3970965	39042	dnaA_2	EDBLDKDH_03947	3960805	3961797	-1	Chromosomal replication initiator protein DnaA
3931923	3970965	39042		EDBLDKDH_03948	3961766	3962569	-1	hypothetical protein
3931923	3970965	39042		EDBLDKDH_03949	3962586	3962813	-1	hypothetical protein
3931923	3970965	39042		EDBLDKDH_03950	3962887	3963084	-1	hypothetical protein
3931923	3970965	39042	recT	EDBLDKDH_03951	3963298	3964101	-1	Protein RecT
3931923	3970965	39042		EDBLDKDH_03952	3964104	3964346	-1	hypothetical protein

3931923	3970965	39042		EDBLDKDH_03953	3964346	3965275	-1	hypothetical protein
3931923	3970965	39042		EDBLDKDH_03954	3965341	3965571	-1	hypothetical protein
3931923	3970965	39042		EDBLDKDH_03955	3965855	3966100	-1	hypothetical protein
3931923	3970965	39042		EDBLDKDH_03956	3966097	3966615	-1	hypothetical protein
3931923	3970965	39042		EDBLDKDH_03957	3966795	3967151	-1	hypothetical protein
3931923	3970965	39042		EDBLDKDH_03958	3967170	3967841	-1	hypothetical protein
3931923	3970965	39042		EDBLDKDH_03959	3967909	3968184	1	hypothetical protein
3931923	3970965	39042		EDBLDKDH_03960	3968138	3968353	-1	hypothetical protein
3931923	3970965	39042	ramB	EDBLDKDH_03961	3968372	3968614	-1	HTH-type transcriptional regulator RamB
3931923	3970965	39042		EDBLDKDH_03962	3968781	3969191	1	hypothetical protein
3931923	3970965	39042		EDBLDKDH_03963	3969299	3969787	1	hypothetical protein
3931923	3970965	39042	xerC_6	EDBLDKDH_03964	3969790	3970965	1	Tyrosine recombinase XerC
3936104	3941877	5773		EDBLDKDH_03915	3934450	3936264	-1	hypothetical protein
3936104	3941877	5773		EDBLDKDH_03916	3936328	3937062	-1	hypothetical protein
3936104	3941877	5773		EDBLDKDH_03917	3937511	3937669	-1	hypothetical protein
3936104	3941877	5773		EDBLDKDH_03918	3937669	3937977	-1	hypothetical protein
3936104	3941877	5773		EDBLDKDH_03919	3937995	3939863	-1	hypothetical protein
3936104	3941877	5773		EDBLDKDH_03920	3939875	3940795	-1	hypothetical protein
3936104	3941877	5773		EDBLDKDH_03921	3940808	3945364	-1	hypothetical protein
3977468	3983847	6379	murI	EDBLDKDH_03974	3977468	3978265	-1	Glutamate racemase
3977468	3983847	6379	slyA_4	EDBLDKDH_03975	3978276	3978728	-1	Transcriptional regulator SlyA
3977468	3983847	6379	gerE	EDBLDKDH_03976	3978803	3979024	-1	Spore germination protein GerE
3977468	3983847	6379		EDBLDKDH_03977	3979212	3979757	-1	hypothetical protein
3977468	3983847	6379	frdB	EDBLDKDH_03978	3979927	3980706	-1	Fumarate reductase iron-sulfur subunit
3977468	3983847	6379	frdA	EDBLDKDH_03979	3980712	3982466	-1	Fumarate reductase flavoprotein subunit
3977468	3983847	6379	sdhC	EDBLDKDH_03980	3982532	3983140	-1	Succinate dehydrogenase cytochrome b558 subunit
3977468	3983847	6379	yslB	EDBLDKDH_03981	3983413	3983847	1	putative protein YslB
4350820	4372441	21621		EDBLDKDH_04360	4350820	4352583	-1	hypothetical protein
4350820	4372441	21621	mutL_2	EDBLDKDH_04361	4352849	4354342	1	DNA mismatch repair protein MutL
4350820	4372441	21621		EDBLDKDH_04362	4354335	4357031	1	hypothetical protein
4350820	4372441	21621		EDBLDKDH_04363	4357028	4358047	1	hypothetical protein
4350820	4372441	21621		EDBLDKDH_04364	4358057	4359877	1	hypothetical protein
4350820	4372441	21621		EDBLDKDH_04365	4360627	4361946	-1	hypothetical protein
4350820	4372441	21621		EDBLDKDH_04366	4361987	4362223	-1	hypothetical protein
4350820	4372441	21621		EDBLDKDH_04367	4362464	4362859	-1	hypothetical protein
4350820	4372441	21621	sinR	EDBLDKDH_04368	4363014	4363355	1	HTH-type transcriptional regulator SinR
4350820	4372441	21621		EDBLDKDH_04369	4364280	4364543	-1	hypothetical protein
4350820	4372441	21621		EDBLDKDH_04370	4364630	4365820	1	hypothetical protein
4350820	4372441	21621		EDBLDKDH_04371	4366137	4366322	-1	hypothetical protein
4350820	4372441	21621		EDBLDKDH_04372	4366755	4367009	1	hypothetical protein
4350820	4372441	21621		EDBLDKDH_04373	4367114	4367812	1	hypothetical protein

4350820	4372441	21621		EDBLDKDH_04374	4368005	4368466	-1	hypothetical protein
4350820	4372441	21621	smpB	EDBLDKDH_04376	4369432	4369899	-1	SsrA-binding protein
4350820	4372441	21621	rnr	EDBLDKDH_04377	4369970	4372441	-1	Ribonuclease R
Lysinibacillus fusiformis strain MGMM7								
**Island start**	**Island end**	**Length**	**Gene ID**	**Locus**	**Gene start**	**Gene end**	**Strand**	**Product**
1928263	1933130	4867	sigV_3	ONFBCBBE_01884	1928767	1929264	1	RNA polymerase sigma factor SigV
1928263	1933130	4867	rsiV_1	ONFBCBBE_01885	1929264	1930163	1	Anti-sigma-V factor RsiV
1928263	1933130	4867	rhaS_5	ONFBCBBE_01886	1930710	1932665	1	HTH-type transcriptional activator RhaS
2107322	2111593	4271		ONFBCBBE_02077	2107531	2109246	-1	hypothetical protein
2107322	2111593	4271		ONFBCBBE_02078	2110528	2110899	1	hypothetical protein
2107322	2111593	4271		ONFBCBBE_02079	2111050	2111514	-1	hypothetical protein
2215552	2255220	39668		ONFBCBBE_02166	2215552	2216013	1	hypothetical protein
2215552	2255220	39668	bltD_2	ONFBCBBE_02167	2216340	2216786	1	Spermine/spermidine acetyltransferase
2215552	2255220	39668		ONFBCBBE_02168	2216915	2217310	1	hypothetical protein
2215552	2255220	39668		ONFBCBBE_02169	2217935	2218711	1	hypothetical protein
2215552	2255220	39668		ONFBCBBE_02170	2219443	2220297	1	hypothetical protein
2215552	2255220	39668	ydjZ	ONFBCBBE_02171	2220347	2220886	1	TVP38/TMEM64 family inner membrane protein YdjZ
2215552	2255220	39668	mco	ONFBCBBE_02172	2221012	2222523	1	Multicopper oxidase mco
2215552	2255220	39668		ONFBCBBE_02173	2223753	2224079	1	hypothetical protein
2215552	2255220	39668	acr3	ONFBCBBE_02174	2224072	2225127	1	Arsenical-resistance protein Acr3
2215552	2255220	39668	arsC	ONFBCBBE_02175	2225139	2225564	1	Arsenate reductase
2215552	2255220	39668		ONFBCBBE_02176	2225599	2225712	1	hypothetical protein
2215552	2255220	39668		ONFBCBBE_02177	2225916	2227274	1	Ferredoxin--NADP reductase
2215552	2255220	39668		ONFBCBBE_02178	2228383	2230689	1	hypothetical protein
2215552	2255220	39668	bluB	ONFBCBBE_02179	2231323	2231964	1	5,6-dimethylbenzimidazole synthase
2215552	2255220	39668		ONFBCBBE_02180	2232265	2232444	1	hypothetical protein
2215552	2255220	39668		ONFBCBBE_02181	2232507	2232692	-1	hypothetical protein
2215552	2255220	39668	serS_2	ONFBCBBE_02182	2233843	2235123	1	Serine--tRNA ligase
2215552	2255220	39668	cmpR_1	ONFBCBBE_02183	2235593	2236462	-1	HTH-type transcriptional activator CmpR
2215552	2255220	39668	pbuE_3	ONFBCBBE_02184	2236563	2237741	1	Purine efflux pump PbuE
2215552	2255220	39668	nudC_1	ONFBCBBE_02185	2238184	2238615	1	NADH pyrophosphatase
2215552	2255220	39668	dapX	ONFBCBBE_02186	2238692	2239126	1	putative N-acetyl-LL-diaminopimelate aminotransferase
2215552	2255220	39668	sdpR_1	ONFBCBBE_02187	2239458	2239769	1	Transcriptional repressor SdpR
2215552	2255220	39668	pbuE_4	ONFBCBBE_02188	2239933	2241090	1	Purine efflux pump PbuE
2215552	2255220	39668		ONFBCBBE_02189	2241318	2241755	-1	hypothetical protein

2215552	2255220	39668		ONFBCBBE_02190	2241760	2242200	-1	putative HTH-type transcriptional regulator
2215552	2255220	39668		ONFBCBBE_02191	2242389	2243018	1	hypothetical protein
2215552	2255220	39668	speG_2	ONFBCBBE_02192	2243405	2243950	1	Spermidine N(1)-acetyltransferase
2215552	2255220	39668		ONFBCBBE_02193	2244300	2244788	1	hypothetical protein
2215552	2255220	39668	yflT	ONFBCBBE_02194	2245034	2245354	-1	General stress protein 17M
2215552	2255220	39668	yybR_1	ONFBCBBE_02195	2245666	2246004	-1	putative HTH-type transcriptional regulator YybR
2215552	2255220	39668	mdaB	ONFBCBBE_02196	2246375	2246926	1	NADPH:quinone oxidoreductase MdaB
2215552	2255220	39668		ONFBCBBE_02197	2246959	2247672	1	putative oxidoreductase
2215552	2255220	39668	xerD_5	ONFBCBBE_02198	2247822	2248562	-1	Tyrosine recombinase XerD
2215552	2255220	39668		ONFBCBBE_02199	2249113	2249931	-1	hypothetical protein
2215552	2255220	39668	adaB_1	ONFBCBBE_02200	2249968	2250501	-1	Methylated-DNA--protein-cysteine methyltransferase, inducible
2215552	2255220	39668	adaA_2	ONFBCBBE_02201	2250485	2251123	-1	Bifunctional transcriptional activator/DNA repair enzyme AdaA
2215552	2255220	39668		ONFBCBBE_02202	2251210	2251923	-1	hypothetical protein
2215552	2255220	39668	alkA_2	ONFBCBBE_02203	2252087	2252998	1	DNA-3-methyladenine glycosylase
2215552	2255220	39668	gltC_2	ONFBCBBE_02204	2253246	2253896	-1	HTH-type transcriptional regulator GltC
2215552	2255220	39668		ONFBCBBE_02205	2253992	2254186	1	hypothetical protein
2215552	2255220	39668		ONFBCBBE_02206	2254627	2255220	-1	hypothetical protein
2235903	2241302	5399	cmpR_1	ONFBCBBE_02183	2235593	2236462	-1	HTH-type transcriptional activator CmpR
2235903	2241302	5399	pbuE_3	ONFBCBBE_02184	2236563	2237741	1	Purine efflux pump PbuE
2235903	2241302	5399	nudC_1	ONFBCBBE_02185	2238184	2238615	1	NADH pyrophosphatase
2235903	2241302	5399	dapX	ONFBCBBE_02186	2238692	2239126	1	putative N-acetyl-LL-diaminopimelate aminotransferase
2235903	2241302	5399	sdpR_1	ONFBCBBE_02187	2239458	2239769	1	Transcriptional repressor SdpR
2235903	2241302	5399	pbuE_4	ONFBCBBE_02188	2239933	2241090	1	Purine efflux pump PbuE
2318275	2322871	4596		ONFBCBBE_02265	2317418	2319838	-1	hypothetical protein
2318275	2322871	4596	yknY_2	ONFBCBBE_02266	2319804	2320511	-1	putative ABC transporter ATP-binding protein YknY
2318275	2322871	4596		ONFBCBBE_02267	2320886	2321089	-1	hypothetical protein
2318275	2322871	4596	yceM	ONFBCBBE_02268	2321305	2322201	1	Putative oxidoreductase YceM
2318275	2322871	4596		ONFBCBBE_02269	2322549	2322770	-1	hypothetical protein
2424983	2431174	6191		ONFBCBBE_02368	2424983	2425513	-1	IS1595 family transposase ISCac2
2424983	2431174	6191		ONFBCBBE_02369	2425615	2426559	1	hypothetical protein
2424983	2431174	6191		ONFBCBBE_02370	2426654	2427691	1	hypothetical protein
2424983	2431174	6191		ONFBCBBE_02371	2427920	2428165	1	hypothetical protein
2424983	2431174	6191		ONFBCBBE_02372	2428361	2428753	-1	hypothetical protein
2424983	2431174	6191		ONFBCBBE_02373	2429531	2429701	-1	hypothetical protein
2424983	2431174	6191		ONFBCBBE_02374	2429861	2430400	-1	queuosine precursor transporter

2424983	2431174	6191	queE_1	ONFBCBBE_02375	2430446	2431174	-1	7-carboxy-7-deazaguanine synthase
2424983	2431174	6191		ONFBCBBE_02376	2431167	2431640	-1	hypothetical protein
2471267	2476590	5323	tdh	ONFBCBBE_02415	2471970	2473001	-1	L-threonine 3-dehydrogenase
2471267	2476590	5323	nemA	ONFBCBBE_02416	2473156	2474226	-1	N-ethylmaleimide reductase
2471267	2476590	5323	bpoC	ONFBCBBE_02417	2474258	2475106	-1	Putative non-heme bromoperoxidase BpoC
2471267	2476590	5323	comR_1	ONFBCBBE_02418	2475187	2475768	-1	HTH-type transcriptional repressor ComR
2471267	2476590	5323		ONFBCBBE_02419	2476078	2476515	-1	hypothetical protein
2529989	2542410	12421		ONFBCBBE_02487	2530869	2532893	1	hypothetical protein
2529989	2542410	12421	xerC_3	ONFBCBBE_02488	2533177	2533515	1	Tyrosine recombinase XerC
2529989	2542410	12421	ruvB_2	ONFBCBBE_02489	2533615	2534712	-1	Holliday junction ATP-dependent DNA helicase RuvB
2529989	2542410	12421		ONFBCBBE_02490	2534713	2536041	-1	hypothetical protein
2529989	2542410	12421		ONFBCBBE_02491	2536051	2536401	-1	hypothetical protein
2529989	2542410	12421		ONFBCBBE_02492	2536427	2538118	-1	hypothetical protein
2529989	2542410	12421		ONFBCBBE_02493	2538139	2538243	-1	hypothetical protein
2529989	2542410	12421	rep_1	ONFBCBBE_02494	2538404	2540149	-1	ATP-dependent DNA helicase Rep
2529989	2542410	12421	recF_3	ONFBCBBE_02495	2540146	2542203	-1	DNA replication and repair protein RecF
2533177	2543908	10731	xerC_3	ONFBCBBE_02488	2533177	2533515	1	Tyrosine recombinase XerC
2533177	2543908	10731	ruvB_2	ONFBCBBE_02489	2533615	2534712	-1	Holliday junction ATP-dependent DNA helicase RuvB
2533177	2543908	10731		ONFBCBBE_02490	2534713	2536041	-1	hypothetical protein
2533177	2543908	10731		ONFBCBBE_02491	2536051	2536401	-1	hypothetical protein
2533177	2543908	10731		ONFBCBBE_02492	2536427	2538118	-1	hypothetical protein
2533177	2543908	10731		ONFBCBBE_02493	2538139	2538243	-1	hypothetical protein
2533177	2543908	10731	rep_1	ONFBCBBE_02494	2538404	2540149	-1	ATP-dependent DNA helicase Rep
2533177	2543908	10731	recF_3	ONFBCBBE_02495	2540146	2542203	-1	DNA replication and repair protein RecF
2533177	2543908	10731		ONFBCBBE_02496	2543036	2543908	-1	hypothetical protein
2549039	2582981	33942	dinB_1	ONFBCBBE_02504	2549039	2550295	-1	DNA polymerase IV
2549039	2582981	33942		ONFBCBBE_02505	2550560	2551237	-1	hypothetical protein
2549039	2582981	33942		ONFBCBBE_02506	2551713	2551832	-1	hypothetical protein
2549039	2582981	33942		ONFBCBBE_02507	2552161	2553027	-1	hypothetical protein
2549039	2582981	33942		ONFBCBBE_02508	2553039	2555603	-1	hypothetical protein
2549039	2582981	33942		ONFBCBBE_02509	2556559	2556909	-1	hypothetical protein
2549039	2582981	33942		ONFBCBBE_02510	2557044	2558039	1	hypothetical protein
2549039	2582981	33942		ONFBCBBE_02511	2558148	2562254	-1	hypothetical protein
2549039	2582981	33942		ONFBCBBE_02512	2562554	2563054	-1	hypothetical protein
2549039	2582981	33942		ONFBCBBE_02513	2563058	2564713	-1	hypothetical protein

2549039	2582981	33942	xerC_4	ONFBCBBE_02514	2564710	2566248	-1	Tyrosine recombinase XerC
2549039	2582981	33942		ONFBCBBE_02515	2566248	2566925	-1	hypothetical protein
2549039	2582981	33942	xerC_5	ONFBCBBE_02516	2566906	2568081	-1	Tyrosine recombinase XerC
2549039	2582981	33942	dinB_2	ONFBCBBE_02517	2568050	2568514	-1	DNA polymerase IV
2549039	2582981	33942		ONFBCBBE_02518	2568511	2568732	-1	hypothetical protein
2549039	2582981	33942		ONFBCBBE_02519	2569094	2569282	1	hypothetical protein
2549039	2582981	33942		ONFBCBBE_02520	2569260	2569697	-1	hypothetical protein
2549039	2582981	33942	nsrR_1	ONFBCBBE_02521	2570020	2570520	1	HTH-type transcriptional repressor NsrR
2549039	2582981	33942	yodC_3	ONFBCBBE_02522	2570623	2571249	-1	Putative NAD(P)H nitroreductase YodC
2549039	2582981	33942	ohrA	ONFBCBBE_02523	2571275	2571679	-1	Organic hydroperoxide resistance protein OhrA
2549039	2582981	33942	sarZ	ONFBCBBE_02524	2571831	2572268	1	HTH-type transcriptional regulator SarZ
2549039	2582981	33942	bmrR_1	ONFBCBBE_02525	2572586	2573404	1	Multidrug-efflux transporter 1 regulator
2549039	2582981	33942	ydfK_2	ONFBCBBE_02526	2573467	2574138	1	putative membrane protein YdfK
2549039	2582981	33942		ONFBCBBE_02527	2574371	2575792	1	hypothetical protein
2549039	2582981	33942		ONFBCBBE_02528	2576065	2577345	-1	hypothetical protein
2549039	2582981	33942		ONFBCBBE_02529	2577671	2577907	-1	hypothetical protein
2549039	2582981	33942		ONFBCBBE_02530	2578046	2578366	1	hypothetical protein
2549039	2582981	33942		ONFBCBBE_02531	2579365	2580279	1	hypothetical protein
2549039	2582981	33942		ONFBCBBE_02532	2580444	2580821	-1	hypothetical protein
2549039	2582981	33942	gerBC_3	ONFBCBBE_02533	2580933	2582177	1	Spore germination protein B3
2549039	2582981	33942	cypM_2	ONFBCBBE_02534	2582226	2582981	-1	Cypemycin N-terminal methyltransferase
2550481	2556573	6092		ONFBCBBE_02505	2550560	2551237	-1	hypothetical protein
2550481	2556573	6092		ONFBCBBE_02506	2551713	2551832	-1	hypothetical protein
2550481	2556573	6092		ONFBCBBE_02507	2552161	2553027	-1	hypothetical protein
2550481	2556573	6092		ONFBCBBE_02508	2553039	2555603	-1	hypothetical protein
2550481	2556573	6092		ONFBCBBE_02509	2556559	2556909	-1	hypothetical protein
2558041	2568176	10135		ONFBCBBE_02511	2558148	2562254	-1	hypothetical protein
2558041	2568176	10135		ONFBCBBE_02512	2562554	2563054	-1	hypothetical protein
2558041	2568176	10135		ONFBCBBE_02513	2563058	2564713	-1	hypothetical protein
2558041	2568176	10135	xerC_4	ONFBCBBE_02514	2564710	2566248	-1	Tyrosine recombinase XerC
2558041	2568176	10135		ONFBCBBE_02515	2566248	2566925	-1	hypothetical protein
2558041	2568176	10135	xerC_5	ONFBCBBE_02516	2566906	2568081	-1	Tyrosine recombinase XerC
2558041	2568176	10135	dinB_2	ONFBCBBE_02517	2568050	2568514	-1	DNA polymerase IV
2618145	2622209	4064		ONFBCBBE_02573	2617734	2618243	1	hypothetical protein
2618145	2622209	4064	azoB	ONFBCBBE_02574	2619331	2620245	-1	NAD(P)H azoreductase

2618145	2622209	4064		ONFBCBBE_02575	2620267	2621043	-1	hypothetical protein
2618145	2622209	4064		ONFBCBBE_02576	2621089	2621277	-1	hypothetical protein
2618145	2622209	4064	hxlR_1	ONFBCBBE_02577	2621527	2621853	1	HTH-type transcriptional activator HxlR
2626179	2637231	11052		ONFBCBBE_02583	2626179	2626313	1	hypothetical protein
2626179	2637231	11052		ONFBCBBE_02584	2626439	2627212	-1	IS3 family transposase ISEnfa5
2626179	2637231	11052	phnT	ONFBCBBE_02585	2627253	2627357	-1	Putative 2-aminoethylphosphonate import ATP-binding protein PhnT
2626179	2637231	11052	gerBA_2	ONFBCBBE_02586	2627848	2628297	1	Spore germination protein B1
2626179	2637231	11052		ONFBCBBE_02587	2629187	2629711	-1	hypothetical protein
2626179	2637231	11052		ONFBCBBE_02588	2630346	2630777	-1	hypothetical protein
2626179	2637231	11052		ONFBCBBE_02589	2632009	2632374	-1	hypothetical protein
2626179	2637231	11052		ONFBCBBE_02590	2632456	2632671	-1	hypothetical protein
2626179	2637231	11052		ONFBCBBE_02591	2632827	2632988	-1	hypothetical protein
2626179	2637231	11052		ONFBCBBE_02592	2633074	2633580	1	hypothetical protein
2626179	2637231	11052		ONFBCBBE_02593	2634354	2634911	-1	IS1595 family transposase ISSpgl1
2626179	2637231	11052		ONFBCBBE_02594	2635022	2635321	-1	hypothetical protein
2626179	2637231	11052		ONFBCBBE_02595	2635594	2635818	1	hypothetical protein
2626179	2637231	11052		ONFBCBBE_02596	2636384	2636662	1	hypothetical protein
2626179	2637231	11052		ONFBCBBE_02597	2636755	2637231	1	hypothetical protein
2841072	2855216	14144	dpp5_2	ONFBCBBE_02788	2841019	2843124	-1	Dipeptidyl-peptidase 5
2841072	2855216	14144	scmP_3	ONFBCBBE_02789	2843235	2844374	-1	N-acetylcysteine deacetylase
2841072	2855216	14144		ONFBCBBE_02790	2844367	2845209	-1	hypothetical protein
2841072	2855216	14144	menC_1	ONFBCBBE_02791	2845257	2846387	-1	o-succinylbenzoate synthase
2841072	2855216	14144	oppF_7	ONFBCBBE_02792	2846422	2847420	-1	Oligopeptide transport ATP-binding protein OppF
2841072	2855216	14144	oppD_8	ONFBCBBE_02793	2847417	2848412	-1	Oligopeptide transport ATP-binding protein OppD
2841072	2855216	14144	gsiD_4	ONFBCBBE_02794	2848432	2849346	-1	Glutathione transport system permease protein GsiD
2841072	2855216	14144	gsiC_5	ONFBCBBE_02795	2849364	2850281	-1	Glutathione transport system permease protein GsiC
2841072	2855216	14144	appA_2	ONFBCBBE_02796	2850347	2851939	-1	Oligopeptide-binding protein AppA
2841072	2855216	14144		ONFBCBBE_02797	2851954	2853444	-1	hypothetical protein
2841072	2855216	14144	dsdA_2	ONFBCBBE_02798	2853747	2855126	-1	D-serine dehydratase
3312422	3318099	5677	murI	ONFBCBBE_03278	3312422	3313219	-1	Glutamate racemase
3312422	3318099	5677	slyA_6	ONFBCBBE_03279	3313230	3313682	-1	Transcriptional regulator SlyA
3312422	3318099	5677	gerE	ONFBCBBE_03280	3313757	3313978	-1	Spore germination protein GerE
3312422	3318099	5677		ONFBCBBE_03281	3314166	3314711	-1	hypothetical protein
3312422	3318099	5677	frdB	ONFBCBBE_03282	3314887	3315666	-1	Fumarate reductase iron-sulfur subunit
3312422	3318099	5677	frdA	ONFBCBBE_03283	3315672	3317426	-1	Fumarate reductase flavoprotein subunit

3312422	3318099	5677	sdhC	ONFBCBBE_03284	3317491	3318099	-1	Succinate dehydrogenase cytochrome b558 subunit
4671760	4683360	11600		ONFBCBBE_04609	4671760	4671951	-1	hypothetical protein
4671760	4683360	11600		ONFBCBBE_04610	4672060	4672230	-1	hypothetical protein
4671760	4683360	11600		ONFBCBBE_04611	4673364	4673822	1	hypothetical protein
4671760	4683360	11600		ONFBCBBE_04612	4674580	4675677	-1	hypothetical protein
4671760	4683360	11600		ONFBCBBE_04613	4675990	4676562	1	hypothetical protein
4671760	4683360	11600		ONFBCBBE_04614	4676566	4678044	1	hypothetical protein
4671760	4683360	11600		ONFBCBBE_04615	4678069	4678311	1	hypothetical protein
4671760	4683360	11600	xerC_7	ONFBCBBE_04616	4678413	4679579	1	Tyrosine recombinase XerC
4671760	4683360	11600	smpB	ONFBCBBE_04618	4680351	4680818	-1	SsrA-binding protein
4671760	4683360	11600	rnr	ONFBCBBE_04619	4680889	4683360	-1	Ribonuclease R
4888091	4893559	5468		ONFBCBBE_04886	4888110	4888475	1	hypothetical protein
4888091	4893559	5468		ONFBCBBE_04887	4888747	4889535	1	hypothetical protein
4888091	4893559	5468		ONFBCBBE_04888	4889998	4891734	1	hypothetical protein
4888091	4893559	5468		ONFBCBBE_04889	4892375	4892764	1	hypothetical protein
4888091	4893559	5468		ONFBCBBE_04890	4892777	4893532	1	hypothetical protein
4888091	4893559	5468		ONFBCBBE_04891	4893533	4894063	1	hypothetical protein
4888110	5021990	133880		ONFBCBBE_04886	4888110	4888475	1	hypothetical protein
4888110	5021990	133880		ONFBCBBE_04887	4888747	4889535	1	hypothetical protein
4888110	5021990	133880		ONFBCBBE_04888	4889998	4891734	1	hypothetical protein
4888110	5021990	133880		ONFBCBBE_04889	4892375	4892764	1	hypothetical protein
4888110	5021990	133880		ONFBCBBE_04890	4892777	4893532	1	hypothetical protein
4888110	5021990	133880		ONFBCBBE_04891	4893533	4894063	1	hypothetical protein
4888110	5021990	133880		ONFBCBBE_04892	4894129	4894581	1	hypothetical protein
4888110	5021990	133880		ONFBCBBE_04893	4894587	4895618	1	hypothetical protein
4888110	5021990	133880		ONFBCBBE_04894	4895618	4896295	1	hypothetical protein
4888110	5021990	133880		ONFBCBBE_04895	4896326	4899337	1	hypothetical protein
4888110	5021990	133880		ONFBCBBE_04896	4899396	4900028	1	hypothetical protein
4888110	5021990	133880		ONFBCBBE_04897	4900121	4900831	1	hypothetical protein
4888110	5021990	133880		ONFBCBBE_04898	4900847	4902694	1	hypothetical protein
4888110	5021990	133880		ONFBCBBE_04899	4902669	4903352	1	hypothetical protein
4888110	5021990	133880		ONFBCBBE_04900	4903440	4903844	1	hypothetical protein
4888110	5021990	133880		ONFBCBBE_04901	4903860	4904657	1	hypothetical protein
4888110	5021990	133880		ONFBCBBE_04902	4904673	4906376	1	hypothetical protein
4888110	5021990	133880		ONFBCBBE_04903	4906474	4914552	1	hypothetical protein

4888110	5021990	133880		ONFBCBBE_04904	4914581	4922647	1	hypothetical protein
4888110	5021990	133880		ONFBCBBE_04905	4922653	4923027	1	hypothetical protein
4888110	5021990	133880		ONFBCBBE_04906	4923039	4923644	1	hypothetical protein
4888110	5021990	133880		ONFBCBBE_04907	4923647	4924153	1	hypothetical protein
4888110	5021990	133880		ONFBCBBE_04908	4924167	4924958	1	hypothetical protein
4888110	5021990	133880		ONFBCBBE_04909	4924980	4929881	1	hypothetical protein
4888110	5021990	133880		ONFBCBBE_04910	4929878	4931683	1	hypothetical protein
4888110	5021990	133880		ONFBCBBE_04911	4931683	4933440	1	hypothetical protein
4888110	5021990	133880		ONFBCBBE_04912	4933443	4935368	1	hypothetical protein
4888110	5021990	133880		ONFBCBBE_04913	4935385	4935825	1	hypothetical protein
4888110	5021990	133880		ONFBCBBE_04914	4935918	4937459	1	hypothetical protein
4888110	5021990	133880		ONFBCBBE_04915	4937517	4938635	1	hypothetical protein
4888110	5021990	133880		ONFBCBBE_04916	4938673	4938966	1	hypothetical protein
4888110	5021990	133880		ONFBCBBE_04917	4939064	4939705	1	hypothetical protein
4888110	5021990	133880		ONFBCBBE_04918	4939722	4941068	1	hypothetical protein
4888110	5021990	133880		ONFBCBBE_04919	4941089	4941742	1	hypothetical protein
4888110	5021990	133880	moaA_2	ONFBCBBE_04920	4941767	4944091	1	GTP 3',8-cyclase
4888110	5021990	133880		ONFBCBBE_04921	4944088	4944669	1	hypothetical protein
4888110	5021990	133880	moaA_3	ONFBCBBE_04922	4944681	4945571	1	GTP 3',8-cyclase
4888110	5021990	133880		ONFBCBBE_04923	4945573	4946700	1	hypothetical protein
4888110	5021990	133880		ONFBCBBE_04924	4946714	4947064	1	hypothetical protein
4888110	5021990	133880		ONFBCBBE_04925	4947151	4949967	1	hypothetical protein
4888110	5021990	133880		ONFBCBBE_04926	4949972	4951906	1	hypothetical protein
4888110	5021990	133880		ONFBCBBE_04927	4952004	4953032	1	hypothetical protein
4888110	5021990	133880		ONFBCBBE_04928	4953050	4954168	1	hypothetical protein
4888110	5021990	133880		ONFBCBBE_04929	4954150	4955109	1	hypothetical protein
4888110	5021990	133880		ONFBCBBE_04930	4955121	4955588	1	hypothetical protein
4888110	5021990	133880		ONFBCBBE_04931	4955588	4956808	1	hypothetical protein
4888110	5021990	133880		ONFBCBBE_04932	4956795	4957016	1	hypothetical protein
4888110	5021990	133880		ONFBCBBE_04933	4957027	4958082	1	hypothetical protein
4888110	5021990	133880		ONFBCBBE_04934	4958492	4959403	1	hypothetical protein
4888110	5021990	133880		ONFBCBBE_04935	4959766	4960494	1	hypothetical protein
4888110	5021990	133880		ONFBCBBE_04936	4960557	4961048	1	hypothetical protein
4888110	5021990	133880		ONFBCBBE_04937	4961060	4961803	1	hypothetical protein
4888110	5021990	133880		ONFBCBBE_04938	4961903	4962643	1	hypothetical protein

4888110	5021990	133880		ONFBCBBE_04939	4962838	4963425	1	hypothetical protein
4888110	5021990	133880		ONFBCBBE_04940	4963451	4964140	1	hypothetical protein
4888110	5021990	133880		ONFBCBBE_04941	4964142	4965221	1	hypothetical protein
4888110	5021990	133880		ONFBCBBE_04942	4965257	4965802	1	hypothetical protein
4888110	5021990	133880		ONFBCBBE_04943	4965802	4966992	1	hypothetical protein
4888110	5021990	133880		ONFBCBBE_04944	4966994	4968439	1	hypothetical protein
4888110	5021990	133880		ONFBCBBE_04945	4968460	4969182	1	hypothetical protein
4888110	5021990	133880		ONFBCBBE_04946	4969261	4969869	1	hypothetical protein
4888110	5021990	133880		ONFBCBBE_04947	4969885	4971186	1	hypothetical protein
4888110	5021990	133880		ONFBCBBE_04948	4971198	4972763	1	hypothetical protein
4888110	5021990	133880		ONFBCBBE_04949	4972794	4974740	1	hypothetical protein
4888110	5021990	133880		ONFBCBBE_04950	4974803	4975570	1	hypothetical protein
4888110	5021990	133880		ONFBCBBE_04951	4975648	4976055	1	hypothetical protein
4888110	5021990	133880	xerC_9	ONFBCBBE_04952	4976828	4977649	1	Tyrosine recombinase XerC
4888110	5021990	133880		ONFBCBBE_04953	4977780	4978133	1	hypothetical protein
4888110	5021990	133880		ONFBCBBE_04954	4978130	4978603	1	hypothetical protein
4888110	5021990	133880		ONFBCBBE_04955	4978791	4979906	1	hypothetical protein
4888110	5021990	133880		ONFBCBBE_04956	4980075	4980677	1	hypothetical protein
4888110	5021990	133880		ONFBCBBE_04957	4980752	4981798	1	hypothetical protein
4888110	5021990	133880		ONFBCBBE_04958	4981870	4982016	1	hypothetical protein
4888110	5021990	133880		ONFBCBBE_04959	4982127	4983437	1	hypothetical protein
4888110	5021990	133880		ONFBCBBE_04960	4983554	4984477	1	hypothetical protein
4888110	5021990	133880		ONFBCBBE_04961	4984477	4985235	1	hypothetical protein
4888110	5021990	133880		ONFBCBBE_04962	4985735	4985833	-1	hypothetical protein
4888110	5021990	133880	rtp	ONFBCBBE_04963	4986043	4986396	-1	Replication termination protein
4888110	5021990	133880		ONFBCBBE_04964	4986499	4987410	-1	hypothetical protein
4888110	5021990	133880		ONFBCBBE_04965	4987485	4988522	-1	hypothetical protein
4888110	5021990	133880		ONFBCBBE_04966	4988593	4989378	-1	hypothetical protein
4888110	5021990	133880		ONFBCBBE_04967	4989708	4990568	-1	hypothetical protein
4888110	5021990	133880		ONFBCBBE_04968	4990626	4991354	-1	hypothetical protein
4888110	5021990	133880		ONFBCBBE_04969	4991382	4992563	-1	hypothetical protein
4888110	5021990	133880		ONFBCBBE_04970	4992565	4993980	-1	hypothetical protein
4888110	5021990	133880		ONFBCBBE_04971	4994275	4997367	-1	hypothetical protein
4888110	5021990	133880		ONFBCBBE_04972	4997367	4997585	-1	hypothetical protein
4888110	5021990	133880		ONFBCBBE_04973	4998099	4998341	1	hypothetical protein

4888110	5021990	133880	soj_2	ONFBCBBE_04974	4998338	4999165	1	Sporulation initiation inhibitor protein Soj
4888110	5021990	133880		ONFBCBBE_04975	4999172	5000155	1	hypothetical protein
4888110	5021990	133880		ONFBCBBE_04976	5000435	5002246	-1	hypothetical protein
4888110	5021990	133880		ONFBCBBE_04977	5002266	5002766	-1	hypothetical protein
4888110	5021990	133880		ONFBCBBE_04978	5002816	5003910	-1	hypothetical protein
4888110	5021990	133880	rep_2	ONFBCBBE_04979	5004094	5004300	-1	ATP-dependent DNA helicase Rep
4888110	5021990	133880		ONFBCBBE_04980	5004792	5005139	-1	hypothetical protein
4888110	5021990	133880		ONFBCBBE_04981	5005374	5005583	-1	hypothetical protein
4888110	5021990	133880		ONFBCBBE_04982	5005641	5006339	-1	hypothetical protein
4888110	5021990	133880		ONFBCBBE_04983	5006354	5006542	-1	hypothetical protein
4888110	5021990	133880		ONFBCBBE_04984	5006641	5006973	-1	hypothetical protein
4888110	5021990	133880		ONFBCBBE_04985	5007520	5008122	-1	Single-stranded DNA-binding protein
4888110	5021990	133880		ONFBCBBE_04986	5008475	5008573	-1	hypothetical protein
4888110	5021990	133880		ONFBCBBE_04987	5009725	5009919	-1	hypothetical protein
4888110	5021990	133880		ONFBCBBE_04988	5009972	5010334	-1	hypothetical protein
4888110	5021990	133880		ONFBCBBE_04989	5010487	5010627	-1	hypothetical protein
4888110	5021990	133880		ONFBCBBE_04991	5011262	5011534	-1	hypothetical protein
4888110	5021990	133880		ONFBCBBE_04992	5011534	5012310	-1	hypothetical protein
4888110	5021990	133880		ONFBCBBE_04993	5012505	5014673	-1	hypothetical protein
4888110	5021990	133880		ONFBCBBE_04994	5014714	5014920	-1	hypothetical protein
4888110	5021990	133880		ONFBCBBE_04995	5015415	5016218	-1	hypothetical protein
4888110	5021990	133880		ONFBCBBE_04996	5017222	5017743	-1	hypothetical protein
4888110	5021990	133880		ONFBCBBE_04997	5020294	5020488	1	hypothetical protein
4888110	5021990	133880		ONFBCBBE_04998	5021344	5021496	-1	hypothetical protein
4888110	5021990	133880		ONFBCBBE_04999	5021598	5021990	-1	hypothetical protein
4894182	4969012	74830		ONFBCBBE_04892	4894129	4894581	1	hypothetical protein
4894182	4969012	74830		ONFBCBBE_04893	4894587	4895618	1	hypothetical protein
4894182	4969012	74830		ONFBCBBE_04894	4895618	4896295	1	hypothetical protein
4894182	4969012	74830		ONFBCBBE_04895	4896326	4899337	1	hypothetical protein
4894182	4969012	74830		ONFBCBBE_04896	4899396	4900028	1	hypothetical protein
4894182	4969012	74830		ONFBCBBE_04897	4900121	4900831	1	hypothetical protein
4894182	4969012	74830		ONFBCBBE_04898	4900847	4902694	1	hypothetical protein
4894182	4969012	74830		ONFBCBBE_04899	4902669	4903352	1	hypothetical protein
4894182	4969012	74830		ONFBCBBE_04900	4903440	4903844	1	hypothetical protein
4894182	4969012	74830		ONFBCBBE_04901	4903860	4904657	1	hypothetical protein

4894182	4969012	74830		ONFBCBBE_04902	4904673	4906376	1	hypothetical protein
4894182	4969012	74830		ONFBCBBE_04903	4906474	4914552	1	hypothetical protein
4894182	4969012	74830		ONFBCBBE_04904	4914581	4922647	1	hypothetical protein
4894182	4969012	74830		ONFBCBBE_04905	4922653	4923027	1	hypothetical protein
4894182	4969012	74830		ONFBCBBE_04906	4923039	4923644	1	hypothetical protein
4894182	4969012	74830		ONFBCBBE_04907	4923647	4924153	1	hypothetical protein
4894182	4969012	74830		ONFBCBBE_04908	4924167	4924958	1	hypothetical protein
4894182	4969012	74830		ONFBCBBE_04909	4924980	4929881	1	hypothetical protein
4894182	4969012	74830		ONFBCBBE_04910	4929878	4931683	1	hypothetical protein
4894182	4969012	74830		ONFBCBBE_04911	4931683	4933440	1	hypothetical protein
4894182	4969012	74830		ONFBCBBE_04912	4933443	4935368	1	hypothetical protein
4894182	4969012	74830		ONFBCBBE_04913	4935385	4935825	1	hypothetical protein
4894182	4969012	74830		ONFBCBBE_04914	4935918	4937459	1	hypothetical protein
4894182	4969012	74830		ONFBCBBE_04915	4937517	4938635	1	hypothetical protein
4894182	4969012	74830		ONFBCBBE_04916	4938673	4938966	1	hypothetical protein
4894182	4969012	74830		ONFBCBBE_04917	4939064	4939705	1	hypothetical protein
4894182	4969012	74830		ONFBCBBE_04918	4939722	4941068	1	hypothetical protein
4894182	4969012	74830		ONFBCBBE_04919	4941089	4941742	1	hypothetical protein
4894182	4969012	74830	moaA_2	ONFBCBBE_04920	4941767	4944091	1	GTP 3',8-cyclase
4894182	4969012	74830		ONFBCBBE_04921	4944088	4944669	1	hypothetical protein
4894182	4969012	74830	moaA_3	ONFBCBBE_04922	4944681	4945571	1	GTP 3',8-cyclase
4894182	4969012	74830		ONFBCBBE_04923	4945573	4946700	1	hypothetical protein
4894182	4969012	74830		ONFBCBBE_04924	4946714	4947064	1	hypothetical protein
4894182	4969012	74830		ONFBCBBE_04925	4947151	4949967	1	hypothetical protein
4894182	4969012	74830		ONFBCBBE_04926	4949972	4951906	1	hypothetical protein
4894182	4969012	74830		ONFBCBBE_04927	4952004	4953032	1	hypothetical protein
4894182	4969012	74830		ONFBCBBE_04928	4953050	4954168	1	hypothetical protein
4894182	4969012	74830		ONFBCBBE_04929	4954150	4955109	1	hypothetical protein
4894182	4969012	74830		ONFBCBBE_04930	4955121	4955588	1	hypothetical protein
4894182	4969012	74830		ONFBCBBE_04931	4955588	4956808	1	hypothetical protein
4894182	4969012	74830		ONFBCBBE_04932	4956795	4957016	1	hypothetical protein
4894182	4969012	74830		ONFBCBBE_04933	4957027	4958082	1	hypothetical protein
4894182	4969012	74830		ONFBCBBE_04934	4958492	4959403	1	hypothetical protein
4894182	4969012	74830		ONFBCBBE_04935	4959766	4960494	1	hypothetical protein
4894182	4969012	74830		ONFBCBBE_04936	4960557	4961048	1	hypothetical protein

4894182	4969012	74830		ONFBCBBE_04937	4961060	4961803	1	hypothetical protein
4894182	4969012	74830		ONFBCBBE_04938	4961903	4962643	1	hypothetical protein
4894182	4969012	74830		ONFBCBBE_04939	4962838	4963425	1	hypothetical protein
4894182	4969012	74830		ONFBCBBE_04940	4963451	4964140	1	hypothetical protein
4894182	4969012	74830		ONFBCBBE_04941	4964142	4965221	1	hypothetical protein
4894182	4969012	74830		ONFBCBBE_04942	4965257	4965802	1	hypothetical protein
4894182	4969012	74830		ONFBCBBE_04943	4965802	4966992	1	hypothetical protein
4894182	4969012	74830		ONFBCBBE_04944	4966994	4968439	1	hypothetical protein
4894182	4969012	74830		ONFBCBBE_04945	4968460	4969182	1	hypothetical protein
4975985	4990191	14206		ONFBCBBE_04951	4975648	4976055	1	hypothetical protein
4975985	4990191	14206	xerC_9	ONFBCBBE_04952	4976828	4977649	1	Tyrosine recombinase XerC
4975985	4990191	14206		ONFBCBBE_04953	4977780	4978133	1	hypothetical protein
4975985	4990191	14206		ONFBCBBE_04954	4978130	4978603	1	hypothetical protein
4975985	4990191	14206		ONFBCBBE_04955	4978791	4979906	1	hypothetical protein
4975985	4990191	14206		ONFBCBBE_04956	4980075	4980677	1	hypothetical protein
4975985	4990191	14206		ONFBCBBE_04957	4980752	4981798	1	hypothetical protein
4975985	4990191	14206		ONFBCBBE_04958	4981870	4982016	1	hypothetical protein
4975985	4990191	14206		ONFBCBBE_04959	4982127	4983437	1	hypothetical protein
4975985	4990191	14206		ONFBCBBE_04960	4983554	4984477	1	hypothetical protein
4975985	4990191	14206		ONFBCBBE_04961	4984477	4985235	1	hypothetical protein
4975985	4990191	14206		ONFBCBBE_04962	4985735	4985833	-1	hypothetical protein
4975985	4990191	14206	rtp	ONFBCBBE_04963	4986043	4986396	-1	Replication termination protein
4975985	4990191	14206		ONFBCBBE_04964	4986499	4987410	-1	hypothetical protein
4975985	4990191	14206		ONFBCBBE_04965	4987485	4988522	-1	hypothetical protein
4975985	4990191	14206		ONFBCBBE_04966	4988593	4989378	-1	hypothetical protein
4975985	4990191	14206		ONFBCBBE_04967	4989708	4990568	-1	hypothetical protein
4992046	5021031	28985		ONFBCBBE_04969	4991382	4992563	-1	hypothetical protein
4992046	5021031	28985		ONFBCBBE_04970	4992565	4993980	-1	hypothetical protein
4992046	5021031	28985		ONFBCBBE_04971	4994275	4997367	-1	hypothetical protein
4992046	5021031	28985		ONFBCBBE_04972	4997367	4997585	-1	hypothetical protein
4992046	5021031	28985		ONFBCBBE_04973	4998099	4998341	1	hypothetical protein
4992046	5021031	28985	soj_2	ONFBCBBE_04974	4998338	4999165	1	Sporulation initiation inhibitor protein Soj
4992046	5021031	28985		ONFBCBBE_04975	4999172	5000155	1	hypothetical protein
4992046	5021031	28985		ONFBCBBE_04976	5000435	5002246	-1	hypothetical protein
4992046	5021031	28985		ONFBCBBE_04977	5002266	5002766	-1	hypothetical protein

4992046	5021031	28985		ONFBCBBE_04978	5002816	5003910	-1	hypothetical protein
4992046	5021031	28985	rep_2	ONFBCBBE_04979	5004094	5004300	-1	ATP-dependent DNA helicase Rep
4992046	5021031	28985		ONFBCBBE_04980	5004792	5005139	-1	hypothetical protein
4992046	5021031	28985		ONFBCBBE_04981	5005374	5005583	-1	hypothetical protein
4992046	5021031	28985		ONFBCBBE_04982	5005641	5006339	-1	hypothetical protein
4992046	5021031	28985		ONFBCBBE_04983	5006354	5006542	-1	hypothetical protein
4992046	5021031	28985		ONFBCBBE_04984	5006641	5006973	-1	hypothetical protein
4992046	5021031	28985		ONFBCBBE_04985	5007520	5008122	-1	Single-stranded DNA-binding protein
4992046	5021031	28985		ONFBCBBE_04986	5008475	5008573	-1	hypothetical protein
4992046	5021031	28985		ONFBCBBE_04987	5009725	5009919	-1	hypothetical protein
4992046	5021031	28985		ONFBCBBE_04988	5009972	5010334	-1	hypothetical protein
4992046	5021031	28985		ONFBCBBE_04989	5010487	5010627	-1	hypothetical protein
4992046	5021031	28985		ONFBCBBE_04991	5011262	5011534	-1	hypothetical protein
4992046	5021031	28985		ONFBCBBE_04992	5011534	5012310	-1	hypothetical protein
4992046	5021031	28985		ONFBCBBE_04993	5012505	5014673	-1	hypothetical protein
4992046	5021031	28985		ONFBCBBE_04994	5014714	5014920	-1	hypothetical protein
4992046	5021031	28985		ONFBCBBE_04995	5015415	5016218	-1	hypothetical protein
4992046	5021031	28985		ONFBCBBE_04996	5017222	5017743	-1	hypothetical protein
4992046	5021031	28985		ONFBCBBE_04997	5020294	5020488	1	hypothetical protein
Lysinibacillus fusiformis strain Cu15								
**Island start**	**Island end**	**Length**	**Gene ID**	**Locus**	**Gene start**	**Gene end**	**Strand**	**Product**
841819	856450	14631	est_1	GNGNAODG_00861	841819	842565	1	Carboxylesterase
841819	856450	14631	rnr	GNGNAODG_00862	842605	845076	1	Ribonuclease R
841819	856450	14631	smpB	GNGNAODG_00863	845147	845614	1	SsrA-binding protein
841819	856450	14631		GNGNAODG_00864	845751	845849	1	hypothetical protein
841819	856450	14631	xerC_1	GNGNAODG_00866	846734	847879	-1	Tyrosine recombinase XerC
841819	856450	14631		GNGNAODG_00867	847933	848382	-1	hypothetical protein
841819	856450	14631		GNGNAODG_00868	848524	848772	1	hypothetical protein
841819	856450	14631		GNGNAODG_00869	848762	849031	1	hypothetical protein
841819	856450	14631		GNGNAODG_00870	849069	850940	1	hypothetical protein
841819	856450	14631		GNGNAODG_00871	851401	851814	1	hypothetical protein
841819	856450	14631		GNGNAODG_00872	852021	852365	1	hypothetical protein
841819	856450	14631		GNGNAODG_00873	852472	852978	1	hypothetical protein
841819	856450	14631		GNGNAODG_00874	853009	853599	1	hypothetical protein
841819	856450	14631		GNGNAODG_00875	853635	853799	1	hypothetical protein
841819	856450	14631		GNGNAODG_00876	853886	854200	1	hypothetical protein

841819	856450	14631		GNGNAODG_00877	854212	854853	1	hypothetical protein
841819	856450	14631		GNGNAODG_00878	854948	855613	1	hypothetical protein
841819	856450	14631		GNGNAODG_00879	856040	856450	1	hypothetical protein
1169444	1178278	8834	dnaB	GNGNAODG_01188	1169444	1170802	1	Replication initiation and membrane attachment protein
1169444	1178278	8834	dnaI	GNGNAODG_01189	1170890	1171759	1	Primosomal protein DnaI
1169444	1178278	8834	thrS	GNGNAODG_01190	1172188	1174119	1	Threonine--tRNA ligase 1
1169444	1178278	8834	infC	GNGNAODG_01191	1174534	1175037	1	Translation initiation factor IF-3
1169444	1178278	8834	rpmI	GNGNAODG_01192	1175069	1175269	1	50S ribosomal protein L35
1169444	1178278	8834	rplT	GNGNAODG_01193	1175325	1175684	1	50S ribosomal protein L20
1169444	1178278	8834		GNGNAODG_01194	1175818	1176081	1	hypothetical protein
1169444	1178278	8834	ysdB	GNGNAODG_01195	1176109	1176501	-1	Sigma-w pathway protein YsdB
1169444	1178278	8834		GNGNAODG_01196	1176607	1177092	1	hypothetical protein
1169444	1178278	8834	ysdC_2	GNGNAODG_01197	1177190	1178278	1	Putative aminopeptidase YsdC
1220355	1228051	7696	lysC	GNGNAODG_01237	1220355	1221581	1	Aspartokinase
1220355	1228051	7696	yslB	GNGNAODG_01238	1221672	1222106	-1	putative protein YslB
1220355	1228051	7696	sdhC	GNGNAODG_01239	1222379	1222987	1	Succinate dehydrogenase cytochrome b558 subunit
1220355	1228051	7696	frdA	GNGNAODG_01240	1223053	1224807	1	Fumarate reductase flavoprotein subunit
1220355	1228051	7696	frdB	GNGNAODG_01241	1224813	1225592	1	Fumarate reductase iron-sulfur subunit
1220355	1228051	7696		GNGNAODG_01242	1225762	1226307	1	hypothetical protein
1220355	1228051	7696	gerE	GNGNAODG_01243	1226495	1226716	1	Spore germination protein GerE
1220355	1228051	7696	slyA_1	GNGNAODG_01244	1226791	1227243	1	Transcriptional regulator SlyA
1220355	1228051	7696	murI	GNGNAODG_01245	1227254	1228051	1	Glutamate racemase
1885226	1896199	10973		GNGNAODG_01894	1885226	1885939	-1	hypothetical protein
1885226	1896199	10973		GNGNAODG_01895	1886198	1886536	1	hypothetical protein
1885226	1896199	10973		GNGNAODG_01896	1886640	1887116	-1	hypothetical protein
1885226	1896199	10973		GNGNAODG_01897	1887209	1887487	-1	hypothetical protein
1885226	1896199	10973		GNGNAODG_01898	1888027	1888251	-1	hypothetical protein
1885226	1896199	10973		GNGNAODG_01899	1888524	1888823	1	hypothetical protein
1885226	1896199	10973		GNGNAODG_01900	1888929	1889486	1	IS1595 family transposase ISSpgl1
1885226	1896199	10973	ydaD	GNGNAODG_01901	1889780	1890781	-1	General stress protein 39
1885226	1896199	10973		GNGNAODG_01902	1890867	1891028	1	hypothetical protein
1885226	1896199	10973		GNGNAODG_01903	1891184	1891399	1	hypothetical protein
1885226	1896199	10973		GNGNAODG_01904	1891482	1891847	1	hypothetical protein
1885226	1896199	10973		GNGNAODG_01905	1892160	1892525	1	hypothetical protein
1885226	1896199	10973		GNGNAODG_01906	1892842	1893555	-1	hypothetical protein

1885226	1896199	10973		GNGNAODG_01907	1893875	1894312	1	hypothetical protein
1885226	1896199	10973		GNGNAODG_01908	1894322	1894453	1	hypothetical protein
1885226	1896199	10973	cwlA	GNGNAODG_01909	1894422	1894667	1	N-acetylmuramoyl-L-alanine amidase CwlA
1885226	1896199	10973		GNGNAODG_01910	1894943	1896199	1	hypothetical protein
1953056	2011987	58931	yhdL	GNGNAODG_01976	1952094	1953074	1	putative anti-sigma-M factor YhdL
1953056	2011987	58931		GNGNAODG_01977	1953056	1953343	1	hypothetical protein
1953056	2011987	58931		GNGNAODG_01978	1953548	1953706	1	hypothetical protein
1953056	2011987	58931		GNGNAODG_01979	1953946	1954668	1	hypothetical protein
1953056	2011987	58931		GNGNAODG_01980	1955071	1955178	-1	hypothetical protein
1953056	2011987	58931		GNGNAODG_01981	1955775	1955978	-1	hypothetical protein
1953056	2011987	58931	mneS_2	GNGNAODG_01982	1956257	1957138	1	Manganese efflux system protein MneS
1953056	2011987	58931		GNGNAODG_01983	1957323	1958147	1	hypothetical protein
1953056	2011987	58931		GNGNAODG_01984	1958229	1958399	-1	hypothetical protein
1953056	2011987	58931		GNGNAODG_01985	1958580	1959224	-1	hypothetical protein
1953056	2011987	58931		GNGNAODG_01986	1959661	1960140	1	hypothetical protein
1953056	2011987	58931		GNGNAODG_01987	1960314	1960625	1	hypothetical protein
1953056	2011987	58931		GNGNAODG_01988	1960694	1961101	1	hypothetical protein
1953056	2011987	58931		GNGNAODG_01989	1961106	1961654	1	hypothetical protein
1953056	2011987	58931		GNGNAODG_01990	1961743	1962459	1	hypothetical protein
1953056	2011987	58931		GNGNAODG_01991	1962592	1962873	1	hypothetical protein
1953056	2011987	58931		GNGNAODG_01992	1963038	1963622	1	hypothetical protein
1953056	2011987	58931		GNGNAODG_01993	1963730	1964137	-1	hypothetical protein
1953056	2011987	58931		GNGNAODG_01994	1964264	1964638	1	hypothetical protein
1953056	2011987	58931		GNGNAODG_01995	1964725	1965666	-1	hypothetical protein
1953056	2011987	58931		GNGNAODG_01996	1966109	1967023	1	hypothetical protein
1953056	2011987	58931		GNGNAODG_01997	1967197	1967838	1	hypothetical protein
1953056	2011987	58931		GNGNAODG_01998	1968070	1968633	-1	hypothetical protein
1953056	2011987	58931		GNGNAODG_01999	1968957	1969115	1	hypothetical protein
1953056	2011987	58931		GNGNAODG_02000	1969443	1969649	1	hypothetical protein
1953056	2011987	58931		GNGNAODG_02001	1969950	1970231	1	hypothetical protein
1953056	2011987	58931		GNGNAODG_02002	1970702	1971007	1	hypothetical protein
1953056	2011987	58931	gbpA	GNGNAODG_02003	1971242	1972600	-1	GlcNAc-binding protein A
1953056	2011987	58931		GNGNAODG_02004	1973491	1973970	1	hypothetical protein
1953056	2011987	58931		GNGNAODG_02005	1973984	1974784	1	hypothetical protein
1953056	2011987	58931		GNGNAODG_02006	1974802	1975323	-1	hypothetical protein

1953056	2011987	58931		GNGNAODG_02007	1975484	1975840	1	hypothetical protein
1953056	2011987	58931		GNGNAODG_02008	1976415	1977134	1	hypothetical protein
1953056	2011987	58931		GNGNAODG_02009	1977820	1978119	1	hypothetical protein
1953056	2011987	58931		GNGNAODG_02010	1978126	1978377	1	hypothetical protein
1953056	2011987	58931	yndE_2	GNGNAODG_02011	1978512	1979642	-1	Spore germination protein YndE
1953056	2011987	58931		GNGNAODG_02012	1979658	1979900	-1	hypothetical protein
1953056	2011987	58931	gerBC_2	GNGNAODG_02013	1979909	1981096	-1	Spore germination protein B3
1953056	2011987	58931	gerBA_2	GNGNAODG_02014	1981124	1982647	-1	Spore germination protein B1
1953056	2011987	58931		GNGNAODG_02015	1982822	1983244	-1	hypothetical protein
1953056	2011987	58931		GNGNAODG_02016	1983673	1983783	1	hypothetical protein
1953056	2011987	58931	xerC_2	GNGNAODG_02017	1983866	1985029	-1	Tyrosine recombinase XerC
1953056	2011987	58931		GNGNAODG_02018	1985140	1985631	-1	hypothetical protein
1953056	2011987	58931	ftsH_1	GNGNAODG_02019	1985851	1986960	1	ATP-dependent zinc metalloprotease FtsH
1953056	2011987	58931		GNGNAODG_02020	1986982	1989270	1	hypothetical protein
1953056	2011987	58931		GNGNAODG_02021	1989302	1989877	-1	hypothetical protein
1953056	2011987	58931		GNGNAODG_02022	1990026	1990274	1	hypothetical protein
1953056	2011987	58931		GNGNAODG_02023	1990336	1990650	1	hypothetical protein
1953056	2011987	58931		GNGNAODG_02024	1990847	1991365	1	hypothetical protein
1953056	2011987	58931		GNGNAODG_02025	1991362	1991607	1	hypothetical protein
1953056	2011987	58931		GNGNAODG_02026	1991889	1992098	1	hypothetical protein
1953056	2011987	58931		GNGNAODG_02027	1992095	1992325	1	hypothetical protein
1953056	2011987	58931		GNGNAODG_02028	1992394	1993287	1	hypothetical protein
1953056	2011987	58931		GNGNAODG_02029	1993238	1994041	1	hypothetical protein
1953056	2011987	58931		GNGNAODG_02030	1994271	1995314	1	hypothetical protein
1953056	2011987	58931	ssbA_2	GNGNAODG_02031	1995311	1995820	1	Single-stranded DNA-binding protein A
1953056	2011987	58931		GNGNAODG_02032	1995843	1996022	1	hypothetical protein
1953056	2011987	58931		GNGNAODG_02033	1996019	1996525	1	hypothetical protein
1953056	2011987	58931		GNGNAODG_02034	1996525	1996689	1	hypothetical protein
1953056	2011987	58931	recU_1	GNGNAODG_02035	1996765	1997280	1	Holliday junction resolvase RecU
1953056	2011987	58931		GNGNAODG_02036	1997533	1997931	1	hypothetical protein
1953056	2011987	58931		GNGNAODG_02037	1998053	1998472	1	hypothetical protein
1953056	2011987	58931		GNGNAODG_02038	1998589	1999866	1	hypothetical protein
1953056	2011987	58931		GNGNAODG_02039	2000388	2001353	1	hypothetical protein
1953056	2011987	58931		GNGNAODG_02040	2001431	2001601	1	hypothetical protein
1953056	2011987	58931		GNGNAODG_02041	2001766	2002218	1	hypothetical protein

1953056	2011987	58931		GNGNAODG_02042	2002360	2002797	1	hypothetical protein
1953056	2011987	58931		GNGNAODG_02043	2002977	2003438	1	hypothetical protein
1953056	2011987	58931		GNGNAODG_02044	2003822	2004634	1	hypothetical protein
1953056	2011987	58931		GNGNAODG_02045	2004621	2005922	1	hypothetical protein
1953056	2011987	58931		GNGNAODG_02046	2005928	2007412	1	hypothetical protein
1953056	2011987	58931		GNGNAODG_02047	2007409	2008425	1	hypothetical protein
1953056	2011987	58931		GNGNAODG_02048	2008568	2008789	1	hypothetical protein
1953056	2011987	58931		GNGNAODG_02049	2008981	2009604	1	hypothetical protein
1953056	2011987	58931		GNGNAODG_02050	2009620	2009979	1	hypothetical protein
1953056	2011987	58931		GNGNAODG_02051	2010000	2011049	1	hypothetical protein
1953056	2011987	58931		GNGNAODG_02052	2011096	2011317	1	hypothetical protein
1953056	2011987	58931		GNGNAODG_02053	2011327	2011677	1	hypothetical protein
1953056	2011987	58931		GNGNAODG_02054	2011655	2011987	1	hypothetical protein
2512097	2516289	4192	rhaS_7	GNGNAODG_02563	2512280	2513869	-1	HTH-type transcriptional activator RhaS
2512097	2516289	4192	yfiY_4	GNGNAODG_02564	2514411	2515409	-1	putative siderophore-binding lipoprotein YfiY
2512097	2516289	4192		GNGNAODG_02565	2516065	2516277	-1	hypothetical protein
2590530	2601758	11228		GNGNAODG_02649	2590530	2590730	1	hypothetical protein
2590530	2601758	11228		GNGNAODG_02650	2591558	2592094	-1	hypothetical protein
2590530	2601758	11228		GNGNAODG_02651	2592403	2593239	-1	hypothetical protein
2590530	2601758	11228	xerD_2	GNGNAODG_02652	2593325	2593522	-1	Tyrosine recombinase XerD
2590530	2601758	11228		GNGNAODG_02653	2593716	2593871	-1	hypothetical protein
2590530	2601758	11228		GNGNAODG_02654	2594055	2594192	-1	hypothetical protein
2590530	2601758	11228		GNGNAODG_02655	2594208	2594828	-1	hypothetical protein
2590530	2601758	11228		GNGNAODG_02656	2594909	2595295	-1	hypothetical protein
2590530	2601758	11228		GNGNAODG_02657	2595497	2595886	1	hypothetical protein
2590530	2601758	11228		GNGNAODG_02658	2595967	2596266	-1	hypothetical protein
2590530	2601758	11228		GNGNAODG_02659	2596430	2596630	-1	hypothetical protein
2590530	2601758	11228		GNGNAODG_02660	2596706	2597002	1	hypothetical protein
2590530	2601758	11228		GNGNAODG_02661	2597008	2597691	-1	hypothetical protein
2590530	2601758	11228	tag	GNGNAODG_02662	2597681	2598256	-1	DNA-3-methyladenine glycosylase 1
2590530	2601758	11228	COQ5_4	GNGNAODG_02663	2598281	2598877	-1	2-methoxy-6-polyprenyl-1,4-benzoquinol methylase, mitochondrial
2590530	2601758	11228		GNGNAODG_02664	2599048	2599278	1	hypothetical protein
2590530	2601758	11228		GNGNAODG_02665	2599293	2599481	1	Aminoglycoside N(6')-acetyltransferase type 1
2590530	2601758	11228		GNGNAODG_02666	2599554	2599745	-1	hypothetical protein
2590530	2601758	11228		GNGNAODG_02667	2600199	2601758	-1	hypothetical protein

4501860	4509610	7750		GNGNAODG_04515	4501860	4503134	1	hypothetical protein
4501860	4509610	7750		GNGNAODG_04516	4504242	4505672	-1	hypothetical protein
4501860	4509610	7750		GNGNAODG_04517	4506149	4507609	1	hypothetical protein
4501860	4509610	7750		GNGNAODG_04518	4508370	4509056	-1	hypothetical protein
4501860	4509610	7750		GNGNAODG_04519	4509143	4509610	1	hypothetical protein
